# ﻿Revision of *Aplosonyx* Chevrolat, 1836 (Coleoptera, Chrysomelidae, Galerucinae) from China, with descriptions of three new species

**DOI:** 10.3897/zookeys.1154.98336

**Published:** 2023-03-21

**Authors:** Chuan Feng, Xing-Ke Yang, Yang Liu, Zhi-Qiang Li

**Affiliations:** 1 Guangdong Public Laboratory of Wild Animal Conservation and Utilization, Guangdong Key Laboratory of Animal Conservation and Resource Utilization, Institute of Zoology, Guangdong Academy of Sciences, Guangzhou, Guangdong 510260, China Institute of Zoology, Guangdong Academy of Sciences Guangdong China; 2 Key Laboratory of Resource Biology and Biotechnology in Western China (Northwest University), Shaanxi Key Laboratory for Animal Conservation, College of Life Science, Northwest University, Taibai North Road 229, Xi’an 710069, China Northwest University Xi’an China; 3 Key Laboratory of Zoological Systematics and Evolution, Institute of Zoology, Chinese Academy of Sciences, Beijing 100101, China Institute of Zoology, Chinese Academy of Sciences Beijing China

**Keywords:** *Aplosonyx*, leaf beetles, new record, new species, taxonomy, China

## Abstract

In this study, 21 species of the leaf-beetle genus *Aplosonyx* in China are described, including three new species, *Aplosonyxancorella***sp. nov.**, *Aplosonyxnigricornis***sp. nov.** and *Aplosonyxwudangensis***sp. nov.**, and 1 new record, *Aplosonyxduvivieri* Jacoby, 1900. Additionally, *Aplosonyxancorafulvescens* Chen, 1964 is elevated to species. A key to the Chinese species of *Aplosonyx* is provided.

## ﻿Introduction

The genus *Aplosonyx* was established by Chevrolat (1836). *Gallerucaalbicornis* (Wiedemann, 1821) was designated as type species by Duponchel and Chevrolat (1842). Although Gistel (1848) emended it to *Haplosonyx*, *Aplosonyx* is regarded as the valid name (Maulik 1936; Gressitt and Kimoto 1963; Kimoto 1989; Yang 1995). *Aplosonyx* is the senior synonym of the genus *Berecyntha* Baly, 1865 synonymized by Chapuis (1875), *Caritheca* Baly, 1877 synonymized by Kimoto (1989) and *Haplonyx* Jacobson, 1895 synonymized by Weise (1924). The genus is distributed in the Oriental Region and southern Palearctic and comprises 60 species worldwide; 21 of them occur in China. The main generic characters for *Aplosonyx* were described by Maulik (1936) and were supplemented by Yang (1995). The species of this genus can be distinguished by the following characters: Head small, frontal tubercles distinct, antennae slender, extend to middle of each elytron, generally three basal antennomeres shining, antennomere 2 the shortest, antennomere 3 longer than antennomere 2, antennomere 4 longest and longer than antennomeres 2 and 3 combined. Pronotum wider than head, nearly 2 × as broad as it is long, basal border not margined, apical border and lateral borders margined; anterior angle thickened, and posterior angle angulated, each corner with large seta-bearing pore; disc of pronotum with transverse depression across the middle. Scutellum triangular, smooth, normally impunctate, in some species, finely covered with punctures. Base of elytra wider than pronotum, humeri are strongly convex, disc is raised, covered with large and deep punctures. Elytral epipleuron broad at the base, gradually narrows from its center, extending to apex of the elytron. Procoxal cavity is closed behind, procoxa is globose. Claws are appendiculate. Male with apex of last visible sternite three lobed; female with the last visible sternite complete.

## ﻿Materials and methods

The morphological characters were examined with an Olympus SZ61 microscope. The genitalia of males from each species were dissected using the following procedure: for dried or ethanol preserved specimens, the abdomen was removed from each specimen, bathed in boiling water for 5–10 minutes, then transferred to a vial containing 10% KOH solution. The abdomen with the aedeagus was washed in distilled water 3 or 4 times, transferred onto a cavity slide using fine forceps and the aedeagus was separated from the abdomen using a hooked, fine dissecting needle.

Habitus images were taken using a Canon 5DSR/Nikon SMZ25 digital camera. Aedeagus images were taken using a Nikon D610 digital camera, attached to a Zeiss V/A1 microscope (with 5× objective lens). A cable shutter release was used to prevent the camera from shaking. To obtain the full depth of focus, all images were stacked using HELICON FOCUS 7 and the resulting output was edited with Adobe Photoshop CC.

### ﻿Abbreviations and depositories used in the paper

**TL** type locality;

**IZGAS** Institute of Zoology, Guangdong Academy of Sciences, Guangzhou, China;

**IZAS**Institute of Zoology, Chinese Academy of Sciences, Beijing, China;

**SYSU**Entomological Collection of Sun Yat-sen University, Guangzhou, China;

**NHMUK**The Natural History Museum, London, UK.

## ﻿Results

*Aplosonyx* is similar to several genera, and a short key to the more closely related genera of *Aplosonyx* in the Hylaspini is provided below.

### ﻿Key to the similar genera of Hylaspini

**Table:** 

1	Anterior metasternal process not extending beyond the front edge of the meso-coxal cavities, basal border of pronotum not margined	**2**
–	Anterior metasternal process extending beyond the front edge of the meso-coxal cavities, pronotum borders margined, disc with or without a pair of transverse depressions	***Gallerucida* Motschulsky, 1860**
2	Anterior and lateral border of pronotum margined, posterior corner of pronotum acute, disc with deep transverse depressions	***Aplosonyx* Chevrolat, 1836**
–	Lateral border of pronotum margined, posterior corner of pronotum rounded, disc without deep transverse depressions	***Sphenoraia* Clark, 1865**

#### ﻿ Aplosonyx

Taxon classificationAnimaliaColeopteraChrysomelidae

Chevrolat, 1836

766BC7CC-2083-55F6-8F6C-9D9DAC1A26B2

Aplosonyx Chevrolat, 1836: 375. Type species: Gallerucaalbicornis Wiedemann, 1821, designated by Duponchel and Chevrolat (1842).
Haplosonyx Gistel, 1848: 14 (emend. for Aplosonyx Chevrolat).
Berecyntha Baly, 1865: 98. Type species: Berecynthatibialis Baly, 1865, original designation. Synonymized by Chapuis (1875: 226).
Caritheca Baly, 1877: 226. Type species: Carithecaquadripustulata Baly, 1877, by monotypy. Synonymized by Kimoto (1989: 169).
Haplonyx Jacobson, 1895: 555 (unjustified emendation). Synonymized by Weise (1924: 147).

##### Distribution.

Oriental Region.

### ﻿Key to Chinese species of *Aplosonyx*

**Table:** 

1	Elytral punctures stronger, interstices of punctures equal to or narrower than diameter of single puncture	**2**
–	Elytral punctures relatively not strong, interstices of punctures wider than diameter of single puncture	**5**
2	Elytron reddish brown with a broad purplish band anterior to middle, which extends forward along suture and expands again on base; in some specimens’ dorsal surface entirely reddish brown	**3**
–	Elytron black with all margins yellow, including the suture	***A.cinctus* Chen, 1964**
3	Elytral punctures close, interstices of punctures narrower than diameter of single puncture; punctures in pronotum close and more in number	**4**
–	Elytral punctures sparse, interstices of punctures equal to diameter of single puncture; punctures in pronotum sparse and fewer in number	***A.fulvescens* Chen, 1964**
4	Abdomen without black spots, elytral surface without wrinkles	***A.ancora* Laboissière, 1934**
–	Abdomen with five pair of black spots, elytral surface somewhat wrinkled	***A.ancorella* sp. nov.**
5	Elytron entirely yellow or yellowish brown	**6**
–	Elytron partly or largely pitchy or metallic	**10**
6	Pronotum black	**7**
–	Pronotum yellowish brown	**8**
7	Elytron reddish brown; elytron with punctures arranged in approximately 10 longitudinal striae	***A.rufipennis* Duvivier, 1892**
–	Elytron pale yellow; elytron with very close punctures, which arranged in approximately 20 irregular longitudinal striae	***A.flavipennis* Chen, 1964**
8	Legs black with femur yellowish brown; abdomen yellowish brown; antennae pitchy black with first antennomere brown	***A.orientalis* Jacoby, 1892**
–	Legs entirely black	**9**
9	Abdomen pitchy black; antennae yellowish brown with apical two or three antennomeres blackish	***A.robinsoni* Jacoby, 1905**
–	Abdomen yellowish brown; antennae black	***A.duvivieri* Jacoby, 1900**
10	Elytron entirely or mostly metallic	**11**
–	Elytron brownish with pitchy markings	**13**
11	Pronotum black, elytron dark bluish green with apex cupreous	***A.emeishanicus* (Lopatin, 2005)**
–	Pronotum brownish, elytron entirely bluish or violaceous	**12**
12	Pronotum with four raised areas in front of transverse furrow; elytron blue or purplish-blue	***A.chalybaeus* (Hope, 1831)**
–	Pronotum without any distinctly raised areas in front of transverse furrow; elytron violaceous or greenish	***A.sublaevicollis* Jacoby, 1889**
13	Pronotum black	**14**
–	Pronotum yellowish brown with black spots	**17**
14	Elytron with blackish band	**15**
–	Elytron with black spots, without blackish band	**16**
15	Elytron with a broad black band at middle, which extends along suture and expands again on base	***A.ornatus* Jacoby, 1892**
–	Elytron with a broad black band at side, which extends along lateral margin of elytron	***A.gansuicus* (Chen, 1942)**
16	Elytron each with 5 black spots	***A.nigriceps* Yang, 1995**
–	Elytron each with 6 black spots	***A.nigricornis* sp. nov.**
17	Pronotum with 3 black spots, elytron with 5 spots	**18**
–	Pronotum with only 1 black spot	**19**
18	Black spots on both sides are large on pronotum	***A.yunlongensis* Jiang, 1992**
–	Black spots on both sides are small on pronotum and almost invisible	***A.wudangensis* sp. nov.**
19	Elytron with 5 spots	***A.omeiensis* Chen, 1942**
–	Elytron with 2 longitudinal strips, 1 or 2 black spots apically	**20**
20	Apex of elytron with 1 spot	***A.pictus* Chen, 1942**
–	Apex of elytron with 2 spots	***A.tianpingshanensis* Yang, 1995**

#### ﻿ Aplosonyx ancora

Taxon classificationAnimaliaColeopteraChrysomelidae

Laboissière, 1934

CD18EE42-BABD-5995-878D-A2960F000A5C

Figs 1A–F , 2A

Aplosonyx ancora Laboissière, 1934: 110.
Aplosonyx ancora ancora : Chen 1964: 204.

##### Specimens examined.

3♂♂2♀♀, China, **Guangdong Province**, Nanling, Chengjia; 720 m a. s. l.; 26 May 2022; Chuang Feng leg.; IZGAS. 1♂2♀♀, China, **Guangdong Province**, same data as for preceding; 31 May 2022; Chuang Feng leg.; IZGAS. 3♂♂4♀♀, China, **Guangdong Province**, Foshan, Lutian; 1 Sept 2021; Zulong Liang leg.; IZGAS. ♀, China, **Guangxi Province**, Jinxiu, Luoxiang; 400 m a. s. l.; 15 May 1999; Decheng Yuan leg.; IZAS; IOZ(E)1566707. ♀, same data as for preceding; 400 m a. s. l.; 15 May 1999; Yanzhou Zhang leg.; IZAS; IOZ(E)1566708. ♀, China, **Guangxi Province**, Napo; 440 m a. s. l.; 7 Apr. 1998; Wenzhu Li leg.; IZAS; IOZ(E)1566709. ♀, China, **Guangxi Province**, Longrui; 20 May 1984; Shimei Song leg.; IZAS; IOZ(E)1566744. ♀, same data as for preceding, IZAS; IOZ(E)1566735. ♂, China, **Guangxi Province**, Longzhou; 200 m a. s. l.; 26 Mar. 1998; Chaodong Zhu leg.; IZAS; IOZ(E)1566713. ♀, China, **Guangxi Province**, Napo, Beidou; 550 m a. s. l.; 12 Apr. 1998 Chunsheng Wu leg.; IZAS; IOZ(E)1566714. ♂, China, **Guangxi Province**, Napo, Beidou; 550 m a. s. l.; 12 Apr. 1998 Chunsheng Wu leg.; IZAS; IOZ(E)1566719. ♂, China, **Guangxi Province**, Napo, Baihe; 440 m a. s. l.; 7 Apr. 1998; Chunsheng Wu leg.; IZAS; IOZ(E)1566715. ♀, China, **Guangxi Province**, Napo, Beidou; 550 m a. s. l.; 12 Apr. 1998 Chunsheng Wu leg.; IZAS; IOZ(E)1566718. ♀, China, **Guangxi Province**, Jinxiu, Luoxiang; 400 m a. s. l.; 14 Apr. 1994; Wenzhu Li leg.; IZAS; IOZ(E)1566717. ♀, same data as for preceding; 15 May 1999; Fusheng Huang leg.; IZAS; IOZ(E)1566720. ♀, same data as for preceding; 15 May 1999; Decheng Yuan leg.; IZAS; IOZ(E)1566721. ♀, China, **Yunnan Province**, Xishuangbanna, Yunjinghong; 710 m a. s. l.; 29 Apr. 1958; Yiran Zhang leg.; IZAS; IOZ(E)1566749. ♀, same data as for preceding; 800 m a. s. l.; 29 Apr. 1958; Leyi Zheng leg.; IZAS; IOZ(E)1566745. ♂, China, **Yunnan Province**, Xishuangbanna, Menga; 18 Apr. 1982; Subai Liao leg.; IOZ(E)1566748.

##### Diagnosis.

This species differs from *A.fulvescens* Chen in the antennae with antennomeres 1–6 yellow and antennomeres 7–11 brown, pronotum and elytron densely covered with large punctures, and the aedeagus apex is distinctly pointed. However, the pronotum and elytron of *A.fulvescens* are sparsely covered with punctures, the antennae are brown with antennomeres 1–3 yellow, and the aedeagus is narrowed in the middle with its apex slightly pointed; in lateral view the apex is strongly bent. This species differs from *A.ancorella* sp. nov. in the abdomen having no black spots, and the interstices of punctures in the elytron not being wrinkled.

**Figure 1.**
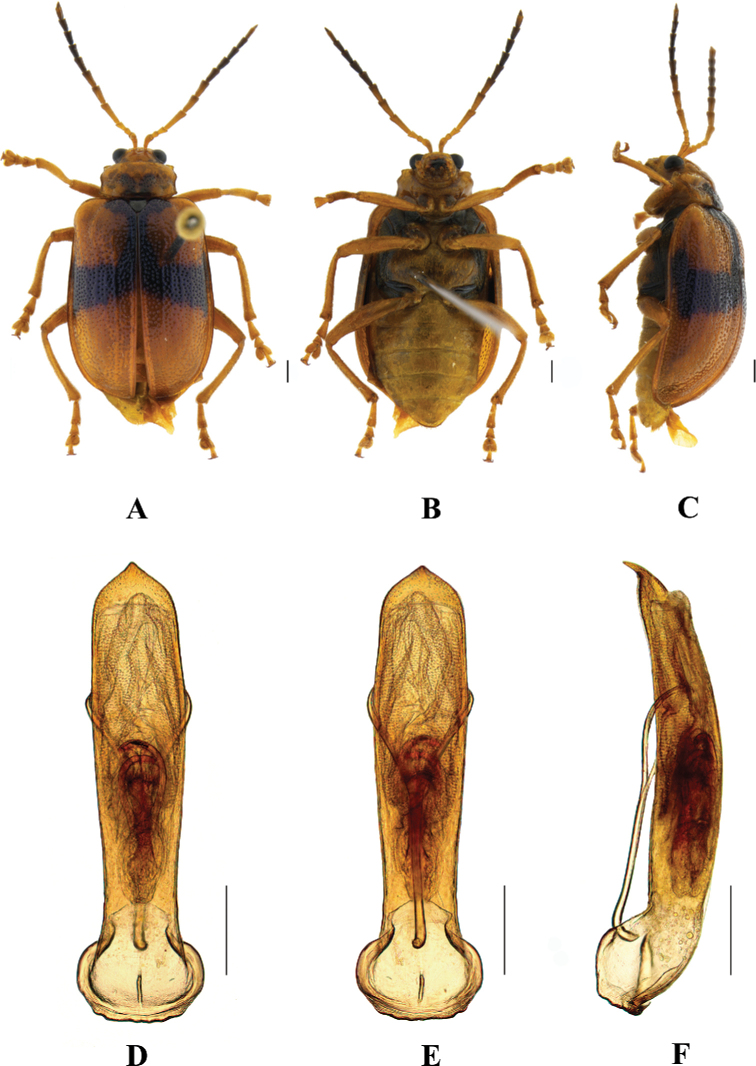
*Aplosonyxancora***A–C** habitus **D–F** aedeagus **A, D** dorsal views **B, E** ventral views **C, F** lateral views. Scale bars 0.5 mm (**D–F**); 1 mm (**A–C**).

**Figure 2.**
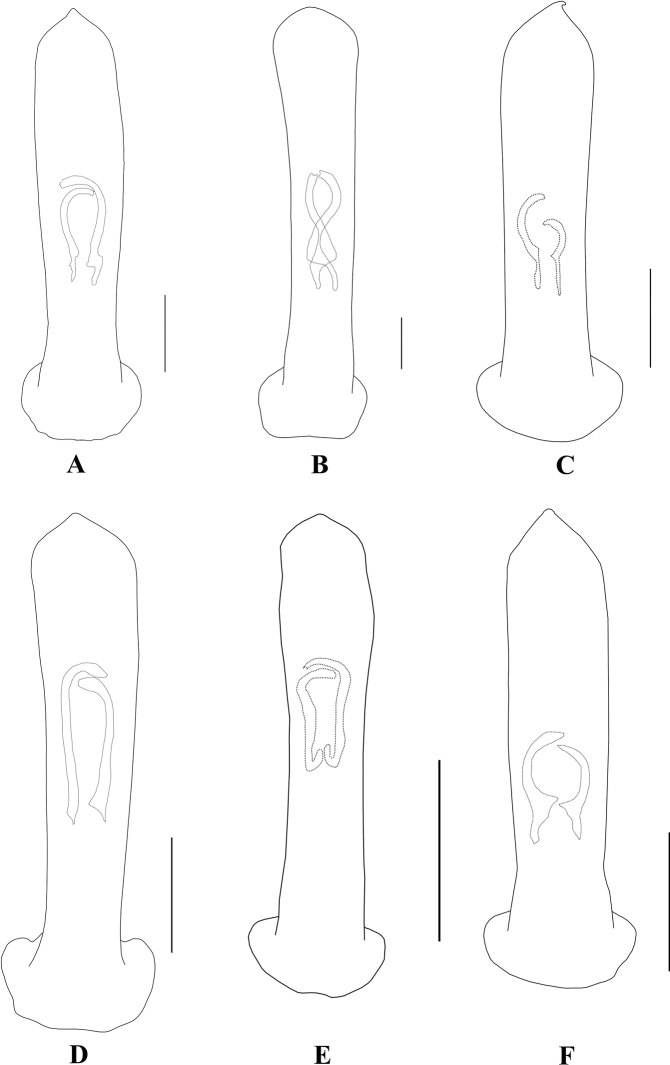
Aedeagus in dorsal view **A***A.ancora***B***A.chalybeus***C***A.cinctus***D***A.duvivieri***F***A.emeishanicus***E***A.flavipennis*. Scale bars: 0.5 mm (**A–E**).

##### Redescription.

**Male.** Length 9.8–12.6 mm, width 5.8–6.6 mm.

Head, pronotum, abdomen, and leg orange, elytra orange or reddish brown, antennae with antennomeres 1–6 yellow and antennomeres 7–11 brown, scutellum black, ventral surface of the thorax black with yellow middle, pronotum with single purple or black spot at base, elytron with a broad purplish band from anterior to middle, extending forward along suture and expanding again on base.

Vertex finely and sparsely covered with punctures. Interocular space 2 × as wide as transverse diameter of eye. Interantennal space 1.3 × as wide as transverse diameter of antennal socket. Frontal tubercles transverse, distinctly raised, each separated by a deep furrow; antennae slender, extended to the middle of the elytra, 0.75 × as long as body; antennomeres 1–3 shiny; antennomeres 4–11 covered with pubescence, antennomere 2 shortest, antennomere 3 longer than antennomere 2, approximately 1.8 × as long as second; antennomere 4 longest, approximately 1.5 × as long as antennomeres 2 and 3 combined; antennomeres 5–10 gradually shortened, shorter than antennomere 4; antennomere 11 slightly longer than antennomere 10, pointed.

Pronotum approximately 2 × as wide as long, lateral border margined, widest at anterior 1/3; disc with transverse furrow, densely covered with large punctures, the interstices of punctures slightly narrower than diameter of punctures, with sparse small punctures in base and apex area of pronotum.

Scutellum triangular, finely covered with punctures.

Elytra wider than pronotum, 0.8 × as long as body, 1.6 × as long as wide, epipleura basally widened, dorsal surface slightly convex, irregularly covered with large and deep punctures, the interstices of punctures narrower than diameter of punctures and lightly covered with small punctures in interstices.

Metasternum 2 × as long as mesosternum. Ventral surface of abdomen with five ventrites, ventrite 1 longest, ventrites 2–4 gradually shortened, apical ventrite slightly longer than ventrite 3, with two subtriangular incisions.

Aedeagus slender, parallel-sided, basally widened, apex pointed, in lateral view slightly bent.

**Female.** Length 9.6–13.2 mm, width 5.6–6.7 mm.

Antennae slightly thinner than in male, antennomere 2 shortest, antennomere 3 approximately 2.5 × as long as second; antennomere 4 longest, 1.2 × as long as antennomeres 2 and 3 combined; apical sternite without incisions.

##### Distribution.

China: Fujian, Guangdong, Guangxi, Yunnan; Vietnam.

#### ﻿ Aplosonyx chalybeus

Taxon classificationAnimaliaColeopteraChrysomelidae

(Hope, 1831)

8FB912F5-60E0-51D6-90C3-8C49ABF0AA29

**Figure 3.**
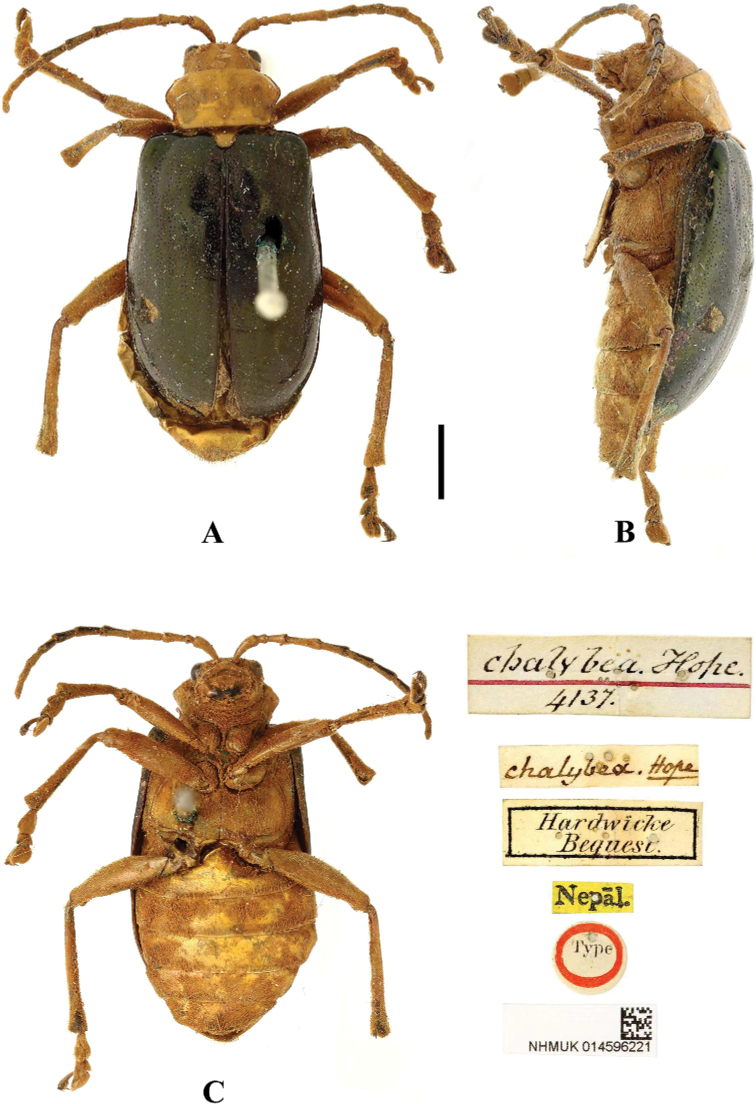
*Aplosonyxchalybeus***A–C** habitus of syntype, NHMUK**A** dorsal view **B** ventral view **C** lateral view. Scale bar: 2 mm (**A–C**).

**Figure 4.**
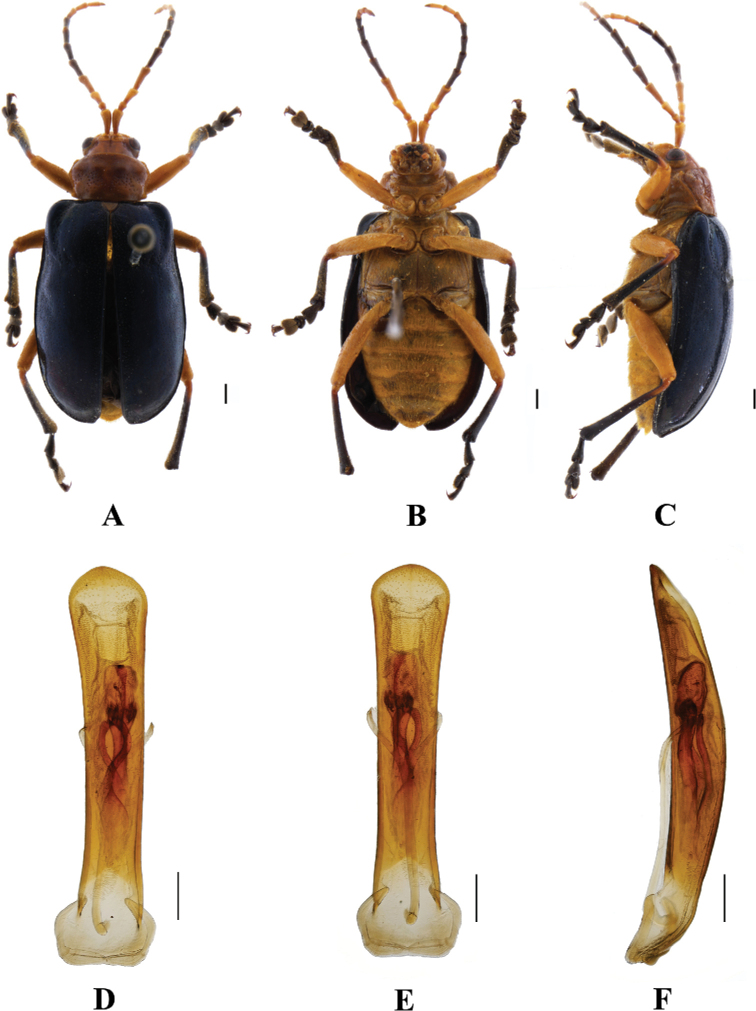
*Aplosonyxchalybeus***A–C** habitus **D–F** aedeagus **A, D** dorsal views **B, E** ventral views **C, F** lateral views. Scale bars 0.5 mm (**D–F**); 1 mm (**A–C**).

Figs 2B , 3A–C , 4A–F

Galleruca chalybea Hope, 1831: 28.
Haplosonyx chalybeus : Duvivier 1892: 440.
Aplosonyx chalibea : Laboissière 1932: 170.
Aplosonyx chalybeus var. Jeanvoinei Laboissière, 1935: 109. Synonymized by Wilcox 1971: 193.

##### Type specimen examined.

♀ ***Syntype*** of *Gallerucachalybea*: *chalybea*. Hope. 4137; Hardwicke Bequest; Nepal. NHMUK014596221.

##### Additional specimens examined.

♀, China, **Yunnan province**, Lushui; 2150 m a. s. l.; 11 Jun. 1981; Subai Liao leg.; IZAS; IOZ(E)1566252. ♂, same data as for preceding; IZAS; IOZ(E)1566219. ♂, same data as for preceding; IZAS; IOZ(E)1566258. ♂, China, **Yunnan province**, Lushui; 1900 m a. s. l.; 8 Jun. 1981; Xuezhong Zhang leg.; IZAS; IOZ(E)1566256. ♂, same data as for preceding; Shuyong Wang leg.; IZAS; IOZ(E)1566257. ♀, China, **Yunnan province**, Ruili; 1100 m a. s. l.; 11 Jun. 1956; Tianrong Huang leg.; IZAS; IOZ(E)1566234. ♀, China, **Yunnan province**, Jinping, Hetouzhai; 1700 m a. s. l.; 12 May 1956; Keren Huang leg.; IOZ(E)1566235. ♀, same data as for preceding; 1600 m a. s. l.; 12 May 1956; Keren Huang leg.; IOZ(E)1566239. ♂, China, **Tibet**, Motuo; 850 m a. s. l.; 14 May 1983; Yinheng Han leg.; IZAS; IOZ(E)1566224. ♂, same data as for preceding; IOZ(E)1566210. ♀, same data as for preceding; IOZ(E)1566211. ♂, same data as for preceding; IOZ(E)1566212. ♂, China, **Tibet**, Motuo, Beibeng; 850 m a. s. l.; 17 May 1983; Yinheng Han leg.; IZAS; IOZ(E)1566213. ♂, same data as for preceding; IOZ(E)1566214. ♀, same data as for preceding; IOZ(E)1566215. ♀, China, **Tibet**, Motuo, Beibeng; 850 m a. s. l.; 24 May 1983; Yinheng Han leg.; IZAS; IOZ(E)1566216. ♂, same data as for preceding; IOZ(E)1566217. ♂, China, **Tibet**, Motuo, 1150 m a. s. l.; 17 Jun. 1983; Yinheng Han leg.; IZAS; IOZ(E)1566209. ♂, China, **Tibet**, Motuo, Xirang; 700 m a. s. l.; 24 Apr. 1983; Yinheng Han leg.; IZAS; IOZ(E)1566218.

##### Diagnosis.

This species can be distinguished from other Chinese species by the yellow antennae with black antennomeres 5–8, the apex of the pronotum with four raised areas, and blue or purplish blue elytra. This species differs from *A.sublaevicollis* in pronotum being widest at anterior 1/3.

##### Redescription.

**Male.** Length 12.2–14.0 mm, width 6.8–7.4 mm.

Head, pronotum, scutellum, and ventral surface of body yellow, antennae yellow with antennomeres 5–8 black, legs black with femur yellow, elytra blue or purplish blue.

Vertex covered with several large punctures; Interocular space 2.6 × as wide as transverse diameter of eye. Interantennal space 1.9 × as wide as transverse diameter of antennal socket. Frontal tubercles transverse, distinctly raised, each separated by a deep furrow; antennae slender, extended to the middle of the elytra, 0.65 × as long as body; antennomeres 1–3 shiny; antennomeres 4–11 covered with pubescence, antennomere 2 shortest, antennomere 3 twice as long as second; antennomere 4 longest, slightly longer than antennomeres 2 and 3 combined; antennomeres 5–10 gradually shortened, shorter than antennomere 4; antennomere 11 slightly longer than antennomere 10, pointed.

Pronotum approximately 1.6 × as wide as long, lateral border margined, widest at anterior 1/3, apex of disc with four raised areas, middle of disc with deep transverse furrow; closely covered with large punctures in furrow and with sparsely small punctures in other parts of pronotum.

Scutellum triangular, finely covered with punctures.

Elytra wider than pronotum, 0.75 × as long as body, 1.9 × as long as wide, with lateral margins straight and subparallel, epipleura basally widened, posteriorly gradually narrowing towards apex, dorsal surface slightly convex, irregularly covered with large punctures, the interstices of punctures wider than diameter of individual punctures, approximately 2 × as wide as diameter of punctures and lightly covered with small punctures in interstices.

Metasternum 2 × as long as mesosternum. Ventral surface of abdomen with five ventrites, ventrite 1 longest, ventrites 2–4 gradually shortened, apical ventrite slightly longer than ventrite 3, with two subtriangular incisions.

Aedeagus slender, parallel-sided, basally widened, apex slightly pointed, in lateral view base and apex slightly bent.

**Female.** Length 12.0–14.2 mm, width 6.6–7.8 mm.

Antennae slightly thinner than in male, antennomere 2 shortest, antennomere 3 approximately 1.5 × as long as second; antennomere 4 longest, 1.4 × as long as antennomeres 2 and 3 combined; apical sternite without incisions.

##### Distribution.

China: Yunnan, Xizang; Vietnam; Myanmar; India; Nepal; Sikkim.

#### ﻿ Aplosonyx cinctus

Taxon classificationAnimaliaColeopteraChrysomelidae

Chen, 1964

DA1C4C93-0CC0-5C94-BEF0-FE887CDDFB07

Figs 2C , 5A–F

Aplosonyx cinctus Chen 1964: 203.

##### Type specimens examined.

***Holotype***: ♀, China, **Yunnan province**, Jinping, Changpotou; 1200 m a. s. l.; 23 May 1956; Keren Huang leg.; IZAS; IOZ(E)215623.

***Paratype***: ♀, same data as for holotype; IOZ(E)215624.

##### Additional specimens examined.

♂, China, **Yunnan province**, Xishuangbanna, Damenglong; 650 m a. s. l.; 4 May 1958; Chunpei Hong leg.; IZAS; IOZ(E)1566412. ♂, same data as for preceding; 6 May 1958; Fuji Pu leg.; IZAS; IOZ(E)1566413. ♂, same data as for preceding; 6 May 1958; Fuji Pu leg.; IZAS; IOZ(E)1566414. ♂, same data as for preceding; 6 May 1958; Shuyong Wang leg.; IZAS; IOZ(E)1566418. ♂, same data as for preceding; 6 Oct. 1958; Zhizi Chen leg.; IZAS; IOZ(E)1566419. ♂, same data as for preceding; 4 May 1958; Chunpei Hong leg.; IZAS; IOZ(E)1566420. ♀, China, **Yunnan province**, Xishuangbanna, Mengzhe; 870 m a. s. l.; 5 Sep. 1958; Fuji Pu leg.; IZAS; IOZ(E)1566421. ♂, China, **Yunnan province**, Xishuangbanna, Damenglong; 650 m a. s. l.; 6 May 1958; Fuji Pu leg.; IZAS; IOZ(E)1566422. ♂, same data as for preceding; Chunpei Hong leg.; IZAS; IOZ(E)1566423. ♂, China, **Yunnan province**, Xishuangbanna, Damenglong; 650 m a. s. l.; 6 May 1958; Leyi Zheng leg.; IZAS; IOZ(E)1566424. ♀, China, **Yunnan province**, Xishuangbanna, Mengzhe; 870 m a. s. l.; 8 Jul. 1958; Shuyong Wang leg.; IZAS; IOZ(E)1566425. ♀, China, **Yunnan province**, Xishuangbanna, Damenglong; 650 m a. s. l.; 6 May 1958; Shuyong Wang leg.; IZAS; IOZ(E)1566393. ♀, same data as for preceding; 4 Oct. 1958; Zhizi Chen leg.; IZAS; IOZ(E)1566395. ♀, same data as for preceding; 4 Oct. 1958; Zhizi Chen leg.; IZAS; IOZ(E)1566396. ♀, China, **Yunnan province**, Xishuangbanna, Mengzhe; 870 m a. s. l.; 10 Jul. 1958; Shuyong Wang leg.; IZAS; IOZ(E)1566397. ♀, same data as for preceding; IOZ(E)1566398. ♀, same data as for preceding; IOZ(E)1566399.

##### Diagnosis.

This species can be distinguished from other congeners by the black pronotum and elytra, all margins of pronotum and elytra yellow, including the yellow suture. This species differs from *A.orientalis* in the color of the body, the slender aedeagus, and in lateral view the apex is strongly bent.

##### Redescription.

**Male.** Length 9.0–10.2 mm, width 5.0–6.0 mm.

Head and abdomen yellow, antennae and ventral surface of thorax black, elytra black with all margins yellow, including the suture of elytra; scutellum black with apex yellow; femur and tibia outside black inside yellow, tarsus and claws brown.

Vertex finely covered with punctures. Interocular space 2.2 × as wide as transverse diameter of eye. Interantennal space 1.7 × as wide as transverse diameter of antennal socket. Frontal tubercles distinctly raised, hook-like, each separated by a deep furrow; antennae slender, 0.7 × as long as body; antennomeres 1–3 shiny; antennomeres 4–11 covered with pubescence, antennomere 2 shortest, antennomere 3 approximately 1.2 × as long as second; antennomere 4 longest, approximately 1.8 × as long as antennomeres 2 and 3 combined; antennomeres 5–10 gradually shortened, shorter than antennomere 4; antennomere 11 slightly longer than antennomere 10, pointed.

Pronotum 2 × as wide as long, lateral border margined, widest at posterior corners, disc with deep transverse furrow, less distinct in middle; closely covered with large punctures in furrow and with sparsely small punctures in other parts of pronotum.

Scutellum triangular, finely covered with punctures.

Elytra wider than pronotum, 0.75 × as long as body, 1.6 × as long as wide, epipleura wide at anterior 1/4, posteriorly gradually narrowing towards apex, dorsal surface slightly convex, irregularly covered with punctures, the interstices of punctures lightly wider than diameter of individual punctures and covered with small punctures in the interstices.

**Figure 5.**
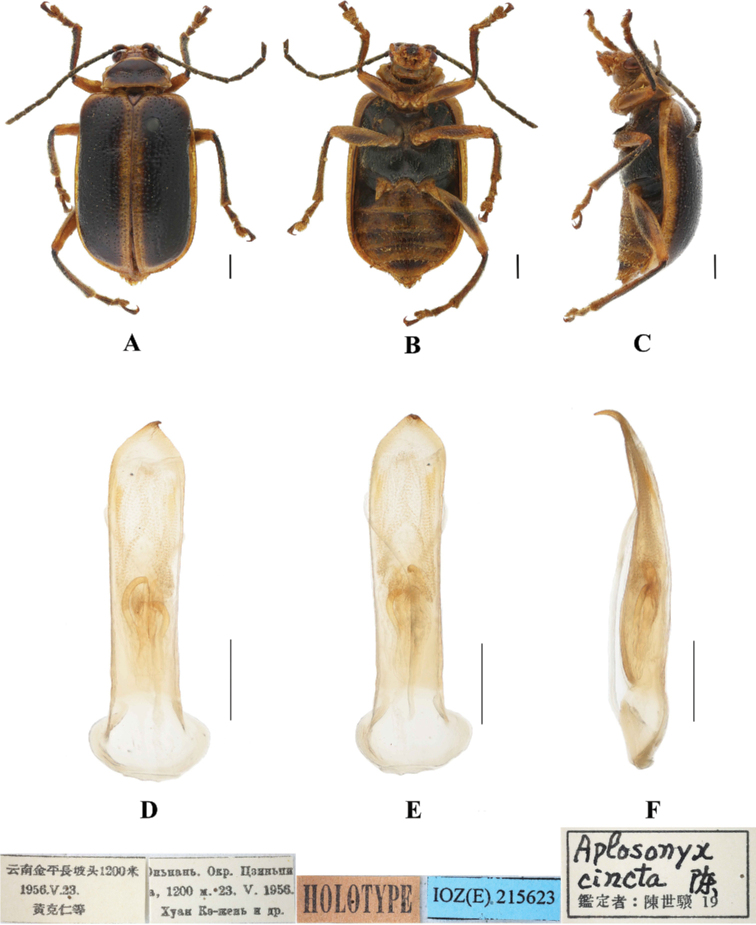
*Aplosonyxcinctus***A–C** habitus of holotype, IZAS**D–F** aedeagus **A, D** dorsal view **B, E** ventral view **C, F** lateral view. Scale bars 0.5 mm (**D–F**); 1 mm (**A–C**).

Metasternum 2 × as long as mesosternum. Ventral surface of abdomen with five ventrites, ventrite 1 longest, ventrites 2–4 gradually shortened, apical ventrite slightly longer than ventrite 3, with two subtriangular incisions.

Aedeagus slender, parallel-sided, slightly narrowed in middle, strongly narrowing in apical tenth, ending in pointed apex, in lateral view apex strongly bent.

**Female.** Length 9.6–10.4 mm, width 5.0–5.8 mm.

Antennae slightly thinner than in male, antennomere 2 shortest, antennomere 3 twice as long as second; antennomere 4 longest, 1.7 × as long as antennomeres 2 and 3 combined; apical sternite without incisions.

##### Distribution.

China: Yunnan.

#### ﻿ Aplosonyx duvivieri

Taxon classificationAnimaliaColeopteraChrysomelidae

Jacoby, 1900 (new record)

A222EEE3-B540-58EC-A5F5-48CA5257F916

Figs 2D , 6 , 7

Haplosonyx duvivieri Jacoby, 1900, 7: 130.
Aplosonyx duvivieri : Maulik 1936: 618.

##### Type specimen examined.

♀, syntype of *Haplosonyxduvivieri* Jacoby; Andrewes Bequest; B.M.1922-221; NHMUK 014596218.

##### Additional specimen examined.

♂, China, **Yunnan Province**, Xishuangbanna, Menga; 29 May. 1958; Shuyong Wang leg.; IOZ(E)1566284.

##### Diagnosis.

This species can be distinguished from other Chinese species by yellow body, antennae, legs, labrum, and mandible black, and dense punctures on the elytra. This species differs from *A.flavipennis* in the head, pronotum, scutellum, and ventral surface of the thorax all being yellow.

##### Redescription.

**Male.** Length 8.9 mm, width 4.4 mm.

Body yellow; antennae, legs, labrum, and mandibles black.

Vertex covered with punctures. Interocular space 2.5 × as wide as transverse diameter of eye. Interantennal space 1.5 × as wide as transverse diameter of antennal socket. Frontal tubercles transverse, each separated by a deep furrow; antennae slender, extended to the middle of the elytra, 0.7 × as long as body; antennomeres 1–3 shiny; antennomeres 4–11 covered with pubescence, antennomere 2 shortest, antennomere 3 approximately 1.8 × as long as second; antennomere 4 longest, approximately 1.5 × as long as antennomeres 2 and 3 combined; antennomeres 5–10 gradually shortened, shorter than antennomere 4; antennomere 11 slightly longer than antennomere 10, pointed.

Pronotum approximately 2 × as wide as long, lateral border margined, widest at posterior corners; disc with deep transverse furrow, covered with large punctures in furrow and with sparsely small punctures in other parts of pronotum.

Scutellum triangular, covered with fine punctures.

Elytra wider than pronotum, 0.7 × as long as body, 1.65 × as long as wide, with lateral margins straight and almost parallel, epipleura wide at anterior 1/4, posteriorly gradually narrowing towards apex, dorsal surface slightly convex, regularly covered with large deep punctures, the interstices of punctures narrower than diameter of punctures, and covered with small punctures in interstices.

**Figure 6.**
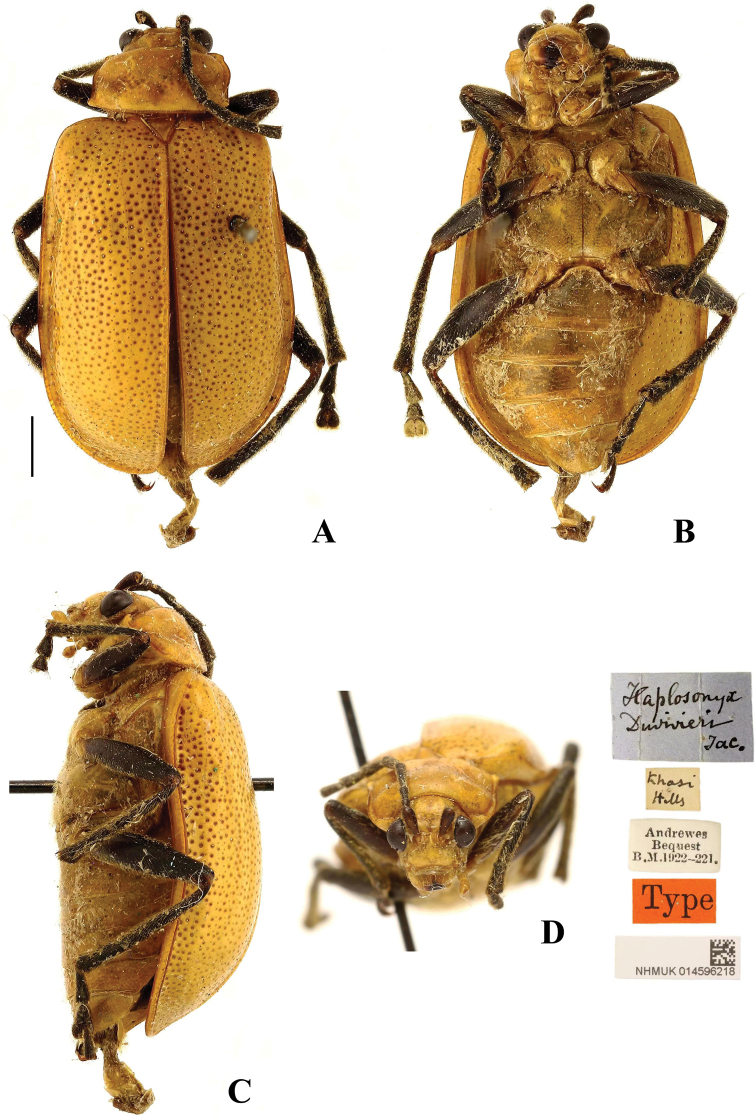
*Aplosonyxduvivieri***A–D** habitus of syntype, NHMUK014596218 **A** dorsal view **B** ventral view **C** lateral view **D** head view. Scale bar: 1 mm (**A–D**).

Metasternum 2 × as long as mesosternum. Ventral surface of abdomen with five ventrites, ventrite 1 longest, ventrites 2–4 gradually shortened, apical ventrite slightly longer than ventrite 3, two subtriangular incisions.

**Figure 7.**
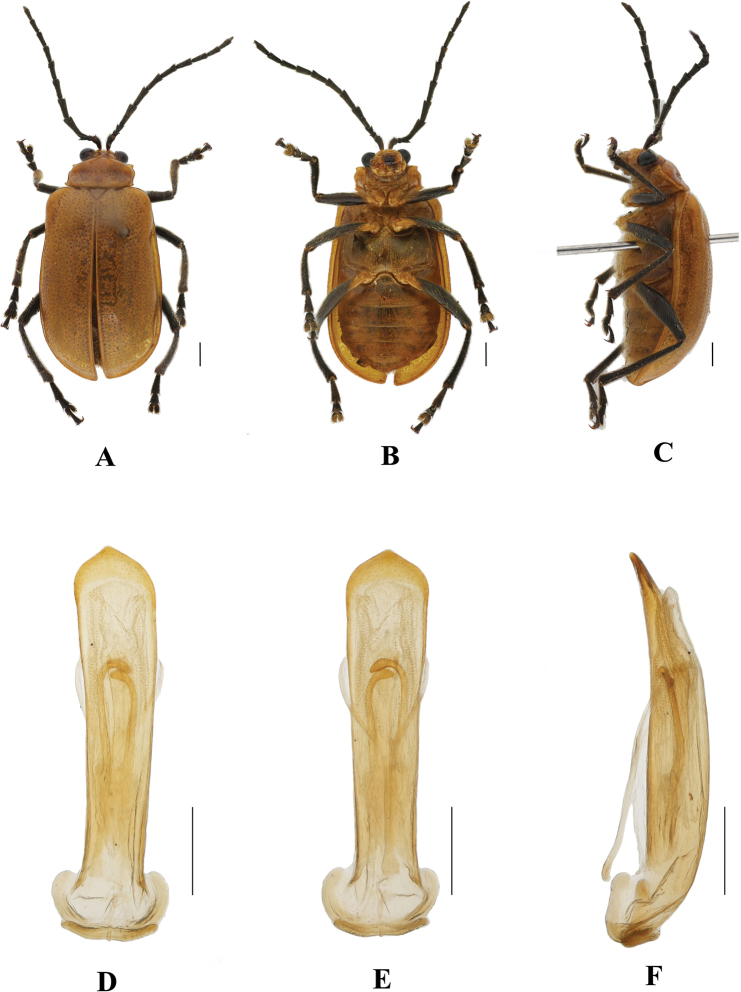
*Aplosonyxduvivieri***A–C** habitus **D–F** aedeagus **A, D** dorsal views **B, E** ventral views **C, F** lateral views. Scale bars 0.5 mm (**D–F**); 1 mm (**A–C**).

Aedeagus slender, parallel-sided, basally widened, narrowed in middle, apex pointed, in lateral view moderately bent.

**Female.** Length 9.4 mm, width 4.7 mm.

Antennae slightly thinner than in male, antennomere 2 shortest, antennomere 3 twice as long as second; antennomere 4 longest, 1.7 × as long as antennomeres 2 and 3 combined; apical sternite without incisions.

##### Distribution.

China: Yunnan; India.

#### ﻿ Aplosonyx emeishanicus

Taxon classificationAnimaliaColeopteraChrysomelidae

(Lopatin, 2005)

D161F5D2-2A91-51E1-AD6B-A3C9D0E149EA

Figs 2E , 8A–F

Gallerucida emeishanica Lopatin, 2005: 877.
Aplosonyx metallicus Chen in Zhang et al. 2008: 65. Synonymized by Bezděk 2012: 383.
Aplosonyx emeishanica : Bezděk 2012: 383.

##### Additional specimens examined.

♂, China, **Sichuan Province**, Mount Emei; 18 Jun. 1955; Keren Huang leg.; IZAS; IOZ(E)1566815. ♀, same data as for preceding; IOZ(E)1566561. ♀, same data as for preceding; IOZ(E)1566576. ♀, same data as for preceding; IOZ(E)1566816. ♂, same data as for preceding; IOZ(E)1566568. ♂, same data as for preceding; IOZ(E)1566571. ♂, same data as for preceding; IOZ(E)1566578. ♂, same data as for preceding; IOZ(E)1566565. ♂, same data as for preceding; IOZ(E)1566566. ♂, same data as for preceding; IOZ(E)1566584. ♂, same data as for preceding; IOZ(E)1566585. ♂, same data as for preceding; IOZ(E)1566586. ♂, China, **Sichuan Province**, Mount Emei; 24 Jun. 1955; Le Wu leg.; IZAS; IOZ(E)1566574. ♂, same data as for preceding; IOZ(E)1566576. ♂, China, **Sichuan Province**, Mount Emei; 1600 m–2100 m a. s. l.; 24 Jun. 1955; Bingrong Ou leg.; IZAS; IOZ(E)1566574. ♀, China, **Sichuan Province**, Mount Emei, Jiulaodong; 1900 m a. s. l.; 22 Jul. 1957; Keren Huang leg.; IZAS; IOZ(E)1566793. ♀, China, **Sichuan Province**, Mount Emei, Jiulaodong; 1800 m a. s. l.; 22 Jul. 1957; Keren Huang leg.; IZAS; IOZ(E)1566794. ♂, China, **Sichuan Province**, Mount Emei, Jiulaodong; 9 Jul. 1957; Zongyuan Wang leg.; IZAS; IOZ(E)1566811. ♀, same data as for preceding; IOZ(E)1566812.

##### Diagnosis.

This species can be distinguished from other Chinese species by the dark bluish green color of the head, pronotum, and elytra.

##### Redescription.

**Male.** Length 4.4–4.8 mm, width 2.6–3.0 mm.

Head, pronotum, scutellum, and ventral surface of body green, antennae and legs brown, elytra dark bluish green with apex cupreous.

Vertex covered with several large punctures. Interocular space 2.3 × as wide as transverse diameter of eye. Interantennal space 1.4 × as wide as transverse diameter of antennal socket. Frontal tubercles transverse, each separated by a deep furrow; antennae slender, 0.85 × as long as body; antennomeres 1–3 shiny; antennomeres 4–11 covered with pubescence, antennomere 2 shortest, antennomere 3 approximately 1.6 × as long as second; antennomere 4 longest, approximately 1.2 × as long as antennomeres 2 and 3 combined; antennomeres 5–10 gradually shortened, shorter than antennomere 4; antennomere 11 slightly longer than antennomere 10, pointed.

**Figure 8.**
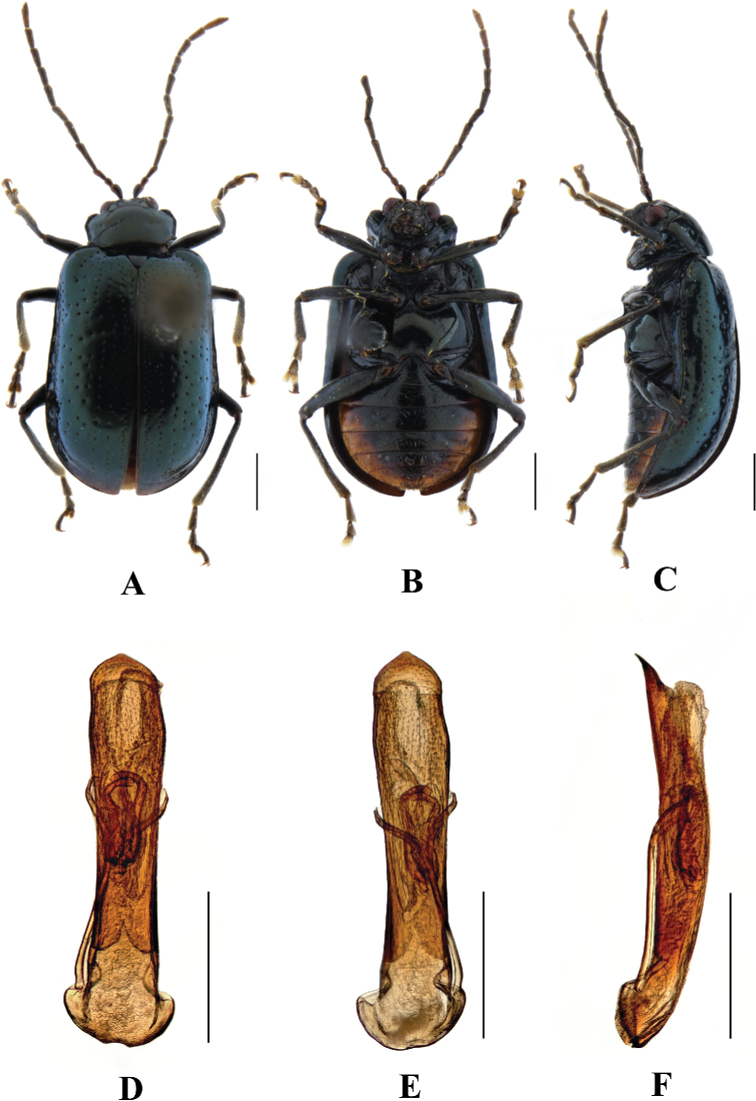
*Aplosonyxemeishanica***A–C** habitus **D–F** aedeagus **A, D** dorsal views **B, E** ventral views **C, F** lateral views. Scale bars 0.5 mm (**D–F**); 1 mm (**A–C**).

Pronotum approximately 1.6 × as wide as long, lateral border margined, widest at anterior 1/3, disc with transverse furrow, finely covered with punctures, only with several large punctures in furrow.

Scutellum triangular, smooth, impunctate.

Elytra wider than pronotum, 0.8 × as long as body, 1.65 × as long as wide, epipleura wide at anterior 1/3, posteriorly gradually narrowing towards apex, dorsal surface slightly convex, regularly covered with punctures, partially arranged in ten rows in each elytron, interstices between punctures approximately 3.5 × as wide as diameter of individual punctures and lightly covered with small punctures.

Metasternum 2 × as long as mesosternum. Ventral surface of abdomen with five ventrites, ventrite 1 longest, ventrites 2–4 gradually shortened, apical ventrite slightly longer than ventrite 3, with two subtriangular incisions.

Aedeagus slender, parallel-sided, basally widened, middle narrowed, apex pointed, in lateral view base and apex slightly bent.

**Female.** Length 4.4–5.0 mm, width 2.5–3.0 mm.

Antennae slightly thinner than in male, antennomere 2 shortest, antennomere 3 approximately 1.2 × as long as second; antennomere 4 longest, 1.5 × as long as antennomeres 2 and 3 combined; apical sternite without incisions.

##### Distribution.

China: Sichuan.

#### ﻿ Aplosonyx flavipennis

Taxon classificationAnimaliaColeopteraChrysomelidae

Chen, 1964

0CD682B8-707A-550F-B7F2-C8A84605A347

Figs 2F , 9A–F

Aplosonyx flavipennis Chen, 1964: 203.

##### Type specimens examined.

***Holotype***: ♂, China, **Yunnan Province**, Xishuangbanna, Menghun; 750 m a. s. l.; 3 Jun. 1958; IZAS; IOZ(E)215625.

***Paratype***: ♂, China, **Yunnan Province**, Xishuangbanna, Menga; 1050 m a. s. l.; 7 Jun. 1958; Shuyong Wang leg.; IZAS; IOZ(E)215627. ♀, China, **Yunnan Province**, Xishuangbanna, Menga; 1050 m a. s. l.; 7 Jun. 1958; Shuyong Wang leg.; IZAS; IOZ(E)215628. ♀, China, **Yunnan Province**, Xishuangbanna, Menga; 1050 m a. s. l.; 7 Jun. 1958; Shuyong Wang leg.; IZAS; IOZ(E)215629.

***Allotype***: ♀, China, **Yunnan Province**, Xishuangbanna, Mengzhe; 870 m a. s. l.; 7 Jul. 1958; Shuyong Wang leg.; IZAS; IOZ(E)215626.

##### Additional specimens examined.

♀, China, **Yunnan Province**, Xishuangbanna, Menga; 1050 m a. s. l.; 7 Jun. 1958; Shuyong Wang leg.; IZAS; IOZ(E)1566487. ♀, China, **Yunnan Province**, Xishuangbanna, Menga; 1050 m a. s. l.; 7 Jun. 1958; Shuyong Wang leg.; IZAS; IOZ(E)1566488. ♀, China, **Yunnan Province**, Xishuangbanna, Menga; 1080 m a. s. l.; 7 Jun. 1958; Shuyong Wang leg.; IZAS; IOZ(E)1566489. ♂, China, **Yunnan Province**, Xishuangbanna, Menga; 1080 m a. s. l.; 11 May 1958; Fuji Pu leg.; IZAS; IOZ(E)1566490. ♂, China, **Yunnan Province**, Xishuangbanna, Menga; 1050 m a. s. l.; 7 May 1958; Chunpei Hong leg.; IZAS; IOZ(E)1566491. ♀, China, **Yunnan Province**, Xishuangbanna, Menga; 1050 m a. s. l.; 7 Jun. 1958; Shuyong Wang leg.; IZAS; IOZ(E)1566492. ♀, China, **Yunnan Province**, Xishuangbanna, Mengzhe; 1200 m a. s. l.; 15 Jun. 1958; Fuji Pu leg.; IZAS; IOZ(E)1566493. ♀, China, **Yunnan Province**, Xishuangbanna, Mengzhe; 1200 m a. s. l.; 15 Jun. 1958; Fuji Pu leg.; IZAS; IOZ(E)1566494. ♀, China, **Yunnan Province**, Xishuangbanna, Menga; 1050 m a. s. l.; 19 Jun. 1958; Fuji Pu leg.; IZAS; IOZ(E)1566496. ♀, China, **Yunnan Province**, Xishuangbanna, Menga; 1000 m a. s. l.; 19 Jun. 1958; Fuji Pu leg.; IZAS; IOZ(E)1566497. ♀, China, **Yunnan Province**, Xishuangbanna, Menga; 1050 m a. s. l.; 7 Jun. 1958; Shuyong Wang leg.; IZAS; IOZ(E)1566498. ♀, China, **Yunnan Province**, Xishuangbanna, Mengzhe; 870 m a. s. l.; 30 Jul. 1958; Shuyong Wang leg.; IZAS; IOZ(E)1566499. ♀, China, **Yunnan Province**, Xishuangbanna, Menga; 1050 m a. s. l.; 17 Oct. 1958; Zhizi Chen leg.; IZAS; IOZ(E)1566500. ♀, China, **Yunnan Province**, Xishuangbanna, Menghun; 750 m a. s. l.; 3 Jun. 1958; Zhizi Chen leg.; IZAS; IOZ(E)1566502. ♀, China, **Yunnan Province**, Xishuangbanna, Menghun; 750 m–950 m a. s. l.; 7 May 1958; Chunpei Hong leg.; IZAS; IOZ(E)1566482. ♂, China, **Yunnan Province**, Xishuangbanna, Menghun; 750 m a. s. l.; 7 May 1958; Chunpei Hong leg.; IZAS; IOZ(E)1566483.

##### Diagnosis.

This species can be distinguished from other Chinese species by its black head and pronotum, and the elytra without any spots. This species differs from *A.duvivieri* in the color of the head, pronotum, scutellum, and ventral surface of thorax being black.

**Figure 9.**
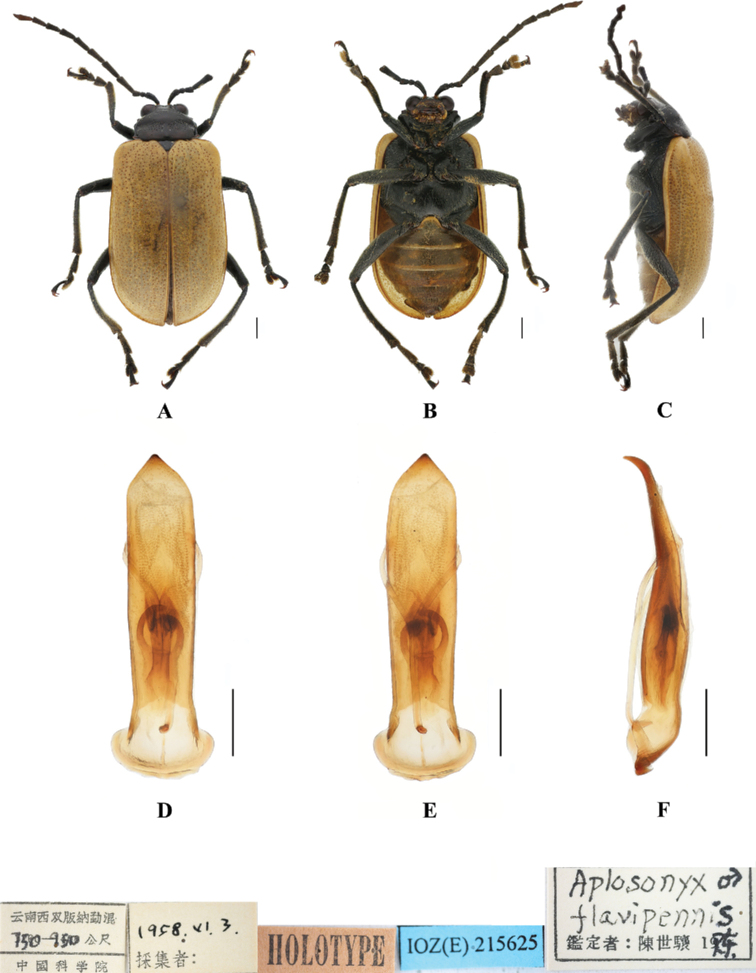
*Aplosonyxflavipennis***A–C** habitus of holotype, IZAS IOZ(E)215625 **D–F** aedeagus **A, D** dorsal views **B, E** ventral views **C, F** lateral views. Scale bars 0.5 mm (**D–F**); 1 mm (**A–C**).

##### Redescription.

**Male.** Length 8.8–10.8 mm, width 4.8–6.0 mm.

Head, antennae, pronotum, scutellum, leg, and ventral surface of thorax black, elytra and abdomen yellow.

Vertex finely covered with punctures. Interocular space 2 × as wide as transverse diameter of eye. Interantennal space 1.5 × as wide as transverse diameter of antennal socket. Frontal tubercles transverse, distinctly raised, each separated by a deep furrow; antennae slender, 0.75 × as long as body; antennomeres 1–3 shiny; antennomeres 4–11 covered with pubescence, antennomere 2 shortest, antennomere 3 approximately 1.4 × as long as second; antennomere 4 longest, approximately 1.8 × as long as antennomeres 2 and 3 combined; antennomeres 5–10 gradually shortened, shorter than antennomere 4; antennomere 11 slightly longer than antennomere 10, pointed.

Pronotum approximately 1.8 × as wide as long, lateral border margined, widest at posterior corners; disc with deep transverse furrow, closely covered with large punctures in furrow and sparsely with small punctures in other parts of pronotum, the interstices of punctures equal to diameter of individual punctures in furrow, and smooth, impunctate in middle of furrow.

Scutellum triangular, finely covered with punctures.

Elytra: wider than pronotum, 0.8 × as long as body, 1.7 × as long as wide, epipleura moderately wide at anterior 1/4, posteriorly gradually narrowing towards apex, dorsal surface slightly convex, regularly covered with large and deep punctures, partially arranged in twenty rows in each elytron, the interstices between punctures wider than the diameter of individual punctures, approximately 2 × as wide as diameter of punctures and lightly covered with small punctures in the interstices.

Metasternum 2 × as long as mesosternum. Ventral surface of abdomen with five ventrites, ventrite 1 longest, ventrites 2–4 gradually shortened, apical ventrite slightly longer than ventrite 4, with two subtriangular incisions.

Aedeagus slender, parallel-sided, basally widened, narrowed in middle, apex distinctly pointed, in lateral view base and apex distinctly bent.

**Female.** Length 9.0–10.2 mm, width 4.8–5.5 mm.

Antennae slightly thinner than in male, antennomere 2 shortest, antennomere 3 1.6 × as long as second; antennomere 4 longest, 2 × as long as antennomeres 2 and 3 combined; apical sternite without incisions.

**Distribution.** China: Yunnan.

#### ﻿ Aplosonyx fulvescens

Taxon classificationAnimaliaColeopteraChrysomelidae

Chen, 1964 stat. nov.

71614BEF-2C87-59C9-B309-9A7420EAD553

Figs 10A–F , 11A

Aplosonyx ancora fulvescens Chen, 1964: 204.

##### Type specimens examined.

***Holotype***: ♂, China, **Hainan Province**; 25 Mar. 1934; IZAS; IOZ(E)215620.

***Paratype***: ♂, same data as for preceding; IOZ(E)215622. ♀, China, **Fujian Province**, Fuzhou; IZAS; IOZ(E)215621.

##### Additional specimens examined.

♀, China, **Hainan Province**, Bawangling; 9 Apr. 1984; IZAS; IOZ(E)1566741. ♀, same data as for preceding; 28 Sep. 1981; IZAS; IOZ(E)1566743. 1♂1♀, China, **Hainan Province**, Jianfengling, Wufeng; 9 May 1981; Shaoying Liang leg.; SYSU. ♀, same data as for preceding, Sanfeng; 26 Aug. 1981; Shaoying Liang leg.; SYSU.

##### Diagnosis.

Chen (1964) described the subspecies *Aplosonyxancorafulvescens* from three specimens collected in Hainan and Fujian. Examination of the type specimen and the additional seven specimens revealed that this subspecies differs from *Aplosonyxancora* in antennae with antennomeres 1–3 yellow and antennomeres 4–11 brown, the pronotum and elytra sparsely covered with small punctures, the apex of the aedeagus slightly pointed, in lateral view apex is bent, the base is wide, and it gradually narrows to the apex. The pronotum and elytra of *Aplosonyxancora* are densely covered with punctures, the antennomeres 1–6 yellow, the aedeagus apex is distinctly pointed. Because these differences are constant among the specimens examined, we elevate the subspecies fulvescens to species level. This species is also similar to *Aplosonyxancorella* sp. nov., which differs in the antennae with antennomeres 1–7 yellow and antennomeres 8–11 brown, the abdomen with five pairs of black spots, and the pronotum and elytra densely covered with large punctures.

##### Redescription.

**Male.** Length 10.6–12.6 mm, width 5.8–6.8 mm.

Head, pronotum, abdomen and leg yellow, elytra reddish brown, antennae brown with antennomeres 1–3 yellow, scutellum black, ventral surface of thorax black with yellow middle, pronotum with 1 small black spot in base, elytra with a broad purplish band from anterior to middle, which extends forward along suture and expends again on base.

Vertex finely covered with punctures. Interocular space 2.2 × as wide as transverse diameter of eye. Interantennal space 1.4 × as wide as transverse diameter of antennal socket. Frontal tubercles transverse, distinctly raised, each separated by a deep furrow; antennae slender, 0.65 × as long as body; antennomeres 1–3 shiny; antennomeres 4–11 covered with pubescence, antennomere 2 shortest, antennomere 3 approximately 1.5 × as long as second; antennomere 4 longest, approximately 1.5 × as long as antennomeres 2 and 3 combined; antennomeres 5–10 gradually shortened, shorter than antennomere 4; antennomere 11 slightly longer than antennomere 10, pointed.

Pronotum approximately 2 × as wide as long, lateral border margined, widest at posterior corners, disc with transverse furrow, sparsely covered with several large punctures, the interstices of punctures equal to diameter of individual punctures.

Scutellum triangular, finely covered with punctures.

Elytra wider than pronotum, 0.78 × as long as body, 1.65 × as long as wide, epipleura wide at anterior 1/4, posteriorly gradually narrowing towards apex, dorsal surface slightly convex, irregularly covered with punctures, the interstices of punctures equal to diameter of individual punctures and lightly covered with small punctures in the interstices.

Metasternum 2 × as long as mesosternum. Ventral surface of abdomen with five ventrites, ventrite 1 longest, ventrites 2–4 gradually shortened, apical ventrite slightly longer than ventrite 3, two subtriangular incisions.

Aedeagus slender, basally widened, apex slightly pointed, in lateral view apex bent.

**Female.** Length 10.8–12.4 mm, width 6.0–6.8 mm.

Antennae slightly thinner than in male, antennomere 2 shortest, antennomere 3 longer than antennomere 2, approximately 1.8 × as long as second; antennomere 4 longest, 1.6 × as long as antennomeres 2 and 3 combined; apical sternite without incisions.

**Figure 10.**
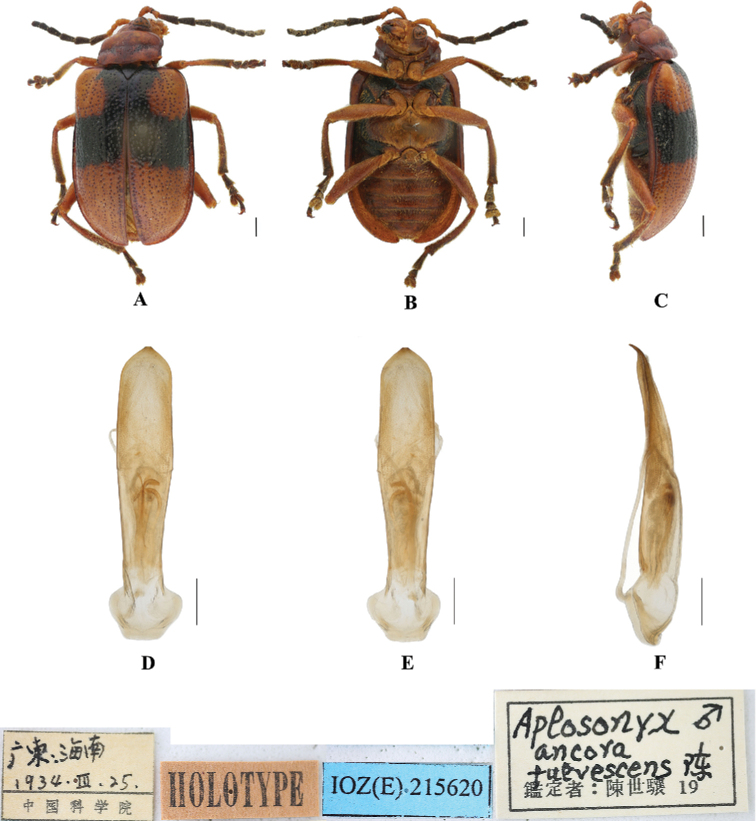
*Aplosonyxfulvescens***A–C** habitus of holotype, IZAS IOZ(E)215620 **D–F** aedeagus **A, D** dorsal views **B, E** ventral views **C, F** lateral views. Scale bars 0.5 mm (**D–F**); 1 mm (**A–C**).

**Figure 11.**
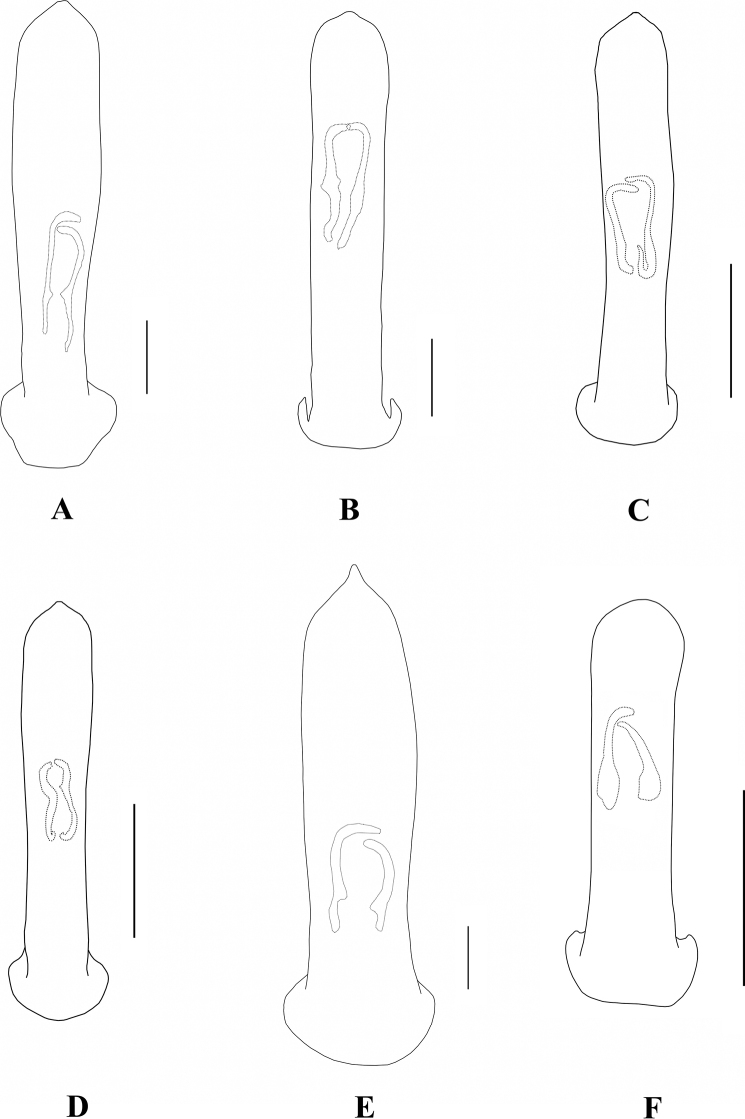
Aedeagus in dorsal view **A***A.fulvescens***B***A.gancuicus***C***A.nigriceps***D***A.omeiensis***E***A.orientalis***F***A.ornatus*. Scale bars: 0.5 mm (**A–E**).

##### Distribution.

China: Fujian, Hainan.

#### ﻿ Aplosonyx gancuicus

Taxon classificationAnimaliaColeopteraChrysomelidae

(Chen, 1942)

477BFB48-B90B-5D40-A743-F675E3C1F3EF

Figs 11B , 12A–F

Galerucida [sic!] gancuica Chen, 1942: 38.
Gallerucida gancuica : Gressitt and Kimoto 1963: 724.
Aplosonyx gancuica : Xu, Nie and Yang 2022: 52.

##### Type specimen examined.

***Holotype***: ♂, China, **Gansu Province**; 8 May 1919; IZAS. IOZ(E)215680.

##### Diagnosis.

This species can be distinguished from other species by the elytra with a broad black band at the side, which extends along the lateral margin of each elytron, and three black spots on each side of the suture.

##### Redescription.

**Male.** Length 6.1 mm, width 3.6 mm.

Head, antennae, pronotum, scutellum, legs, and ventral surface of body black, elytra yellow, each elytron with a broad black band and three black spots.

Vertex sparsely covered with punctures. Interocular space 2 × as wide as transverse diameter of eye. Interantennal space 1.8 × as wide as transverse diameter of antennal socket. Frontal tubercles transverse and raised, each separated by a deep furrow; antennae slender, antennomeres 1–3 shiny; antennomeres 4–8 covered with pubescence, antennomere 2 shortest, antennomere 3 approximately 1.5 × as long as second; antennomere 4 longest, approximately 1.5 × as long as antennomeres 2 and 3 combined; antennomeres 5–8 gradually shortened, shorter than antennomere 4.

Pronotum 1.8 × as wide as long, lateral border margined, widest at posterior corners, disc with sparse punctures.

Scutellum triangular, finely covered with punctures.

Elytra: wider than pronotum, 0.7 × as long as body, 1.6 × as long as wide, epipleura moderately wide at anterior 1/3, posteriorly gradually narrowing towards apex, dorsal surface slightly convex, covered with large deep punctures, partially arranged in ten rows in each elytron, the interstices between punctures wider than diameter of individual punctures, approximately 2 × as wide as the diameter of individual punctures and lightly covered with small punctures in interstices.

**Figure 12.**
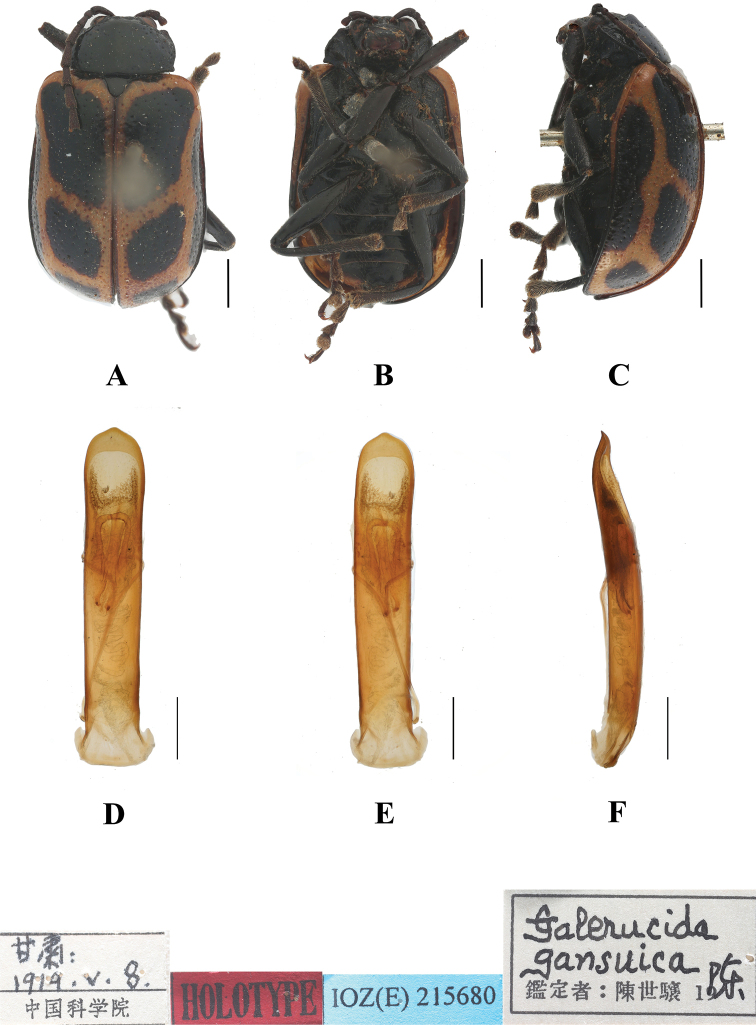
*Aplosonyxgansuica***A–C** habitus of holotype, IZAS IOZ(E)215680 **D–F** aedeagus **A, D** dorsal views **B, E** ventral views **C, F** lateral views. Scale bars 0.5 mm (**D–F**); 1 mm (**A–C**).

Metasternum 2 × as long as mesosternum. Ventral surface of abdomen with five ventrites, ventrite 1 longest, ventrites 2–4 gradually shortened, apical ventrite slightly longer than ventrite 3, with two subtriangular incisions.

Aedeagus slender, parallel-sided, apically pointed, in lateral view moderately wavy in apex.

##### Distribution.

China: Gansu.

#### ﻿ Aplosonyx nigriceps

Taxon classificationAnimaliaColeopteraChrysomelidae

Yang, 1995

DF5CFFAD-2D71-5826-BFB0-5A39889CBA29

Figs 11C , 13A–F

Aplosonyx nigriceps Yang, 1995: 91.

##### Type specimens examined.

***Holotype***: ♂, China, **Hubei Province**, Lichuan; 1300 m a. s. l.; 23 Jul. 1989; Shuyong Wang leg.; IZAS.

***Allotype***: ♀, China, **Hubei Province**, Lichuan, Xingdou Mt; 810 m a. s. l.; 22 Jul. 1989; Shuyong Wang leg.; IZAS.

***Paratype***: 1♂1♀, China, **Hubei Province**, Lichuan; 1300 m a. s. l.; 23 Jul. 1989; Shuyong Wang leg.; IZAS. ♂, China, **Hubei Province**, Lichuan, Xingdou Mt; 1100 m a. s. l.; 22 Jul. 1989; Shuyong Wang leg.; IZAS. 2♀♀, China, **Hubei Province**, Lichuan, Xingdou Mt; 1100 m a. s. l.; 22 Jul. 1989; Shuyong Wang leg.; IZAS. ♂, China, **Hubei Province**, Hefeng, shayuan; 1300 m a. s. l.; 1 Aug. 1989; Shuyong Wang leg.; IZAS. ♀, China, **Hubei Province**, Badong; 1700 m a. s. l.; 21 May 1989; Wenzhen Ma leg.; IZAS.

##### Diagnosis.

This species can be distinguished from other species by its black pronotum, and each elytron with five black spots: one at the base near the scutellum, a pair in the middle, and apically two spots which are connected. This species differs from *A.omeiensis* in having a black head and pronotum, the abdomen yellowish brown, and the shape of the spots on the elytra.

##### Redescription.

**Male.** Length 4.5–5.0 mm, width 2.7–3.2 mm.

Head, pronotum and scutellum black, antennae, ventral surface of the thorax and legs brown, abdomen yellowish brown, elytra yellow, each elytron with five black spots, one at base near scutellum, a pair in the middle, and a pair of apical spots which are connected.

Vertex finely and sparsely covered with punctures. Interocular space 2.1 × as wide as transverse diameter of eye. Interantennal space 1.5 × as wide as transverse diameter of antennal socket. Frontal tubercles transverse, each separated by a deep furrow; antennae slender, 0.75 × as long as body; antennomeres 1–3 shiny; antennomeres 4–11 covered with pubescence, antennomeres 2 and 3 shortest, antennomere 3 nearly equal in length and shape to antennomere 2, antennomere 4 longest, approximately 1.5 × as long as antennomeres 2 and 3 combined; antennomeres 5–10 gradually shortened, shorter than antennomere 4; antennomere 11 slightly longer than 10, pointed.

**Figure 13.**
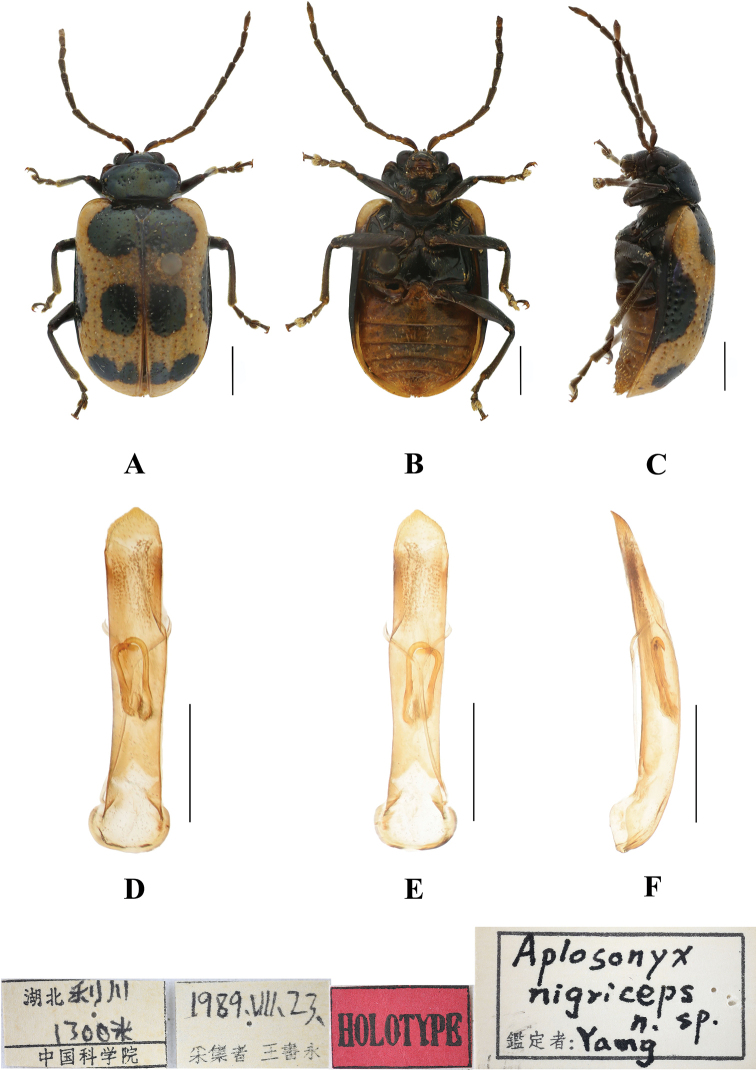
*Aplosonyxnigriceps***A–C** habitus of holotype, IZAS**D–F** aedeagus **A, D** dorsal views **B, E** ventral views **C, F** lateral views. Scale bars 0.5 mm (**D–F**); 1 mm (**A–C**).

Pronotum 1.9 × as wide as long, lateral border margined, widest at anterior 1/3; disc with deep transverse furrow, less distinct in middle; closely covered with large punctures in furrow and with sparsely small punctures in other parts of pronotum.

Scutellum triangular, finely covered with punctures.

Elytra: wider than pronotum, 0.7 × as long as body, 1.5 × as long as wide, epipleura wide at anterior 1/3, posteriorly gradually narrowing towards apex, dorsal surface slightly convex, regularly covered with large and deep punctures, partially arranged in ten rows in each elytron, the interstices between punctures wider than diameter of individual punctures, approximately 2.5 × as wide as the diameter of individual punctures and lightly covered with small punctures in interstices.

Metasternum 2 × as long as mesosternum. Ventral surface of abdomen with five ventrites, ventrite 1 longest, ventrites 2–4 gradually shortened, apical ventrite slightly longer than ventrite 3, with two subtriangular incisions.

Aedeagus slender, parallel-sided, basally widened, apically pointed, in lateral view moderately bent.

**Female.** Length 4.6–5.0 mm, width 2.8–3.2 mm.

Antennae slightly thinner than in male, without short hairs, antennomere 4 longest, approximately 1.2 × as long as antennomeres 2 and 3 combined; apical sternite without incisions.

##### Distribution.

China: Hubei, Sichuan.

#### ﻿ Aplosonyx omeiensis

Taxon classificationAnimaliaColeopteraChrysomelidae

Chen, 1942

193921CE-D3E4-5EB6-9220-59C7637D789A

Figs 11D , 14A–F

Aplosonyx pictus omeiensis Chen, 1942: 40.
Aplosonyx omeiensis : Zhang et al. 2008: 65. Raised from Aplosonyxpictusomeiensis Chen.

##### Type specimens examined.

***Paratypes***: 2♂♂3♀♀, China, **Sichuan Province**, Mount Emei, Sep. 1912; IZAS.

##### Additional specimens examined.

♂, China, **Sichuan Province**, Mount Emei, Jiulaodong; 1800 m a. s. l.; 7 Jul. 1957; Fuxing Zhu leg.; IZAS. ♂, China, **Sichuan Province**, Mount Emei, Jiulaodong; 1800 m a. s. l.; 16 Jun. 1957; Youcai Yu leg.; IZAS. ♂, China, **Sichuan Province**, Mount Emei, Jiulaodong; 1800 m a. s. l.; 7 Jul. 1957; Zongyuan Wang leg.; IZAS; IOZ(E)1566650. ♂, China, **Sichuan Province**, Mount Emei, 17 Jun. 1955; Keren Huang leg.; IZAS; IOZ(E)1566630. ♀, China, **Sichuan Province**, Mount Emei, 17 Jun. 1955; Keren Huang leg.; IZAS; IOZ(E)1566632. ♀, China, **Sichuan Province**, Mount Emei, 17 Jun. 1955; Keren Huang leg.; IZAS; IOZ(E)1566639. ♂, China, **Sichuan Province**, Mount Emei, 17 Jun. 1955; Keren Huang leg.; IZAS; IOZ(E)1566647. ♂, China, **Sichuan Province**, Mount Emei, 1800 m a. s. l.; 24 Jun. 1955; Bingrong Ou leg.; IZAS; IOZ(E)1566634. ♂, China, **Sichuan Province**, Mount Emei, 1800 m a. s. l.; 23 Jun. 1955; Bingrong Ou leg.; IZAS; IOZ(E)1566635. ♂, China, **Sichuan Province**, Mount Emei, 2100 m a. s. l.; 24 Jun. 1955; Bingrong Ou leg.; IZAS; IOZ(E)1566636.

##### Diagnosis.

This species can be distinguished from other species by the pronotum with a black spot, each elytron with five black spots, the middle and apex with one pair of spots, and one spot at the base. This species differs from *A.nigriceps* in having a black abdomen with pale margins.

##### Redescription.

**Male.** Length 4.6–4.8 mm, width 2.6–2.8 mm.

Head, antennae, pronotum, elytra and leg yellow, vertex, scutellum and ventral surface of the body black, abdomen with pale margins, pronotum with a black spot in middle, each elytron with five black spots, middle and apex with one pair of spots, and base with one spot.

Vertex finely and sparsely covered with punctures. Interocular space 2 × as wide as transverse diameter of eye. Interantennal space 1.5 × as wide as transverse diameter of antennal socket. Frontal tubercles transverse, each separated by a deep furrow; antennae slender, 0.7 × as long as body; antennomeres 1–3 shiny; antennomeres 4–11 covered with pubescence, antennomere 2 shortest, antennomere 3 twice as long as second; antennomere 4 longest, approximately 1.2 × as long as antennomeres 2 and 3 combined; antennomeres 5–10 gradually shortened, shorter than antennomere 4; antennomere 11 slightly longer than antennomere 10, pointed.

**Figure 14.**
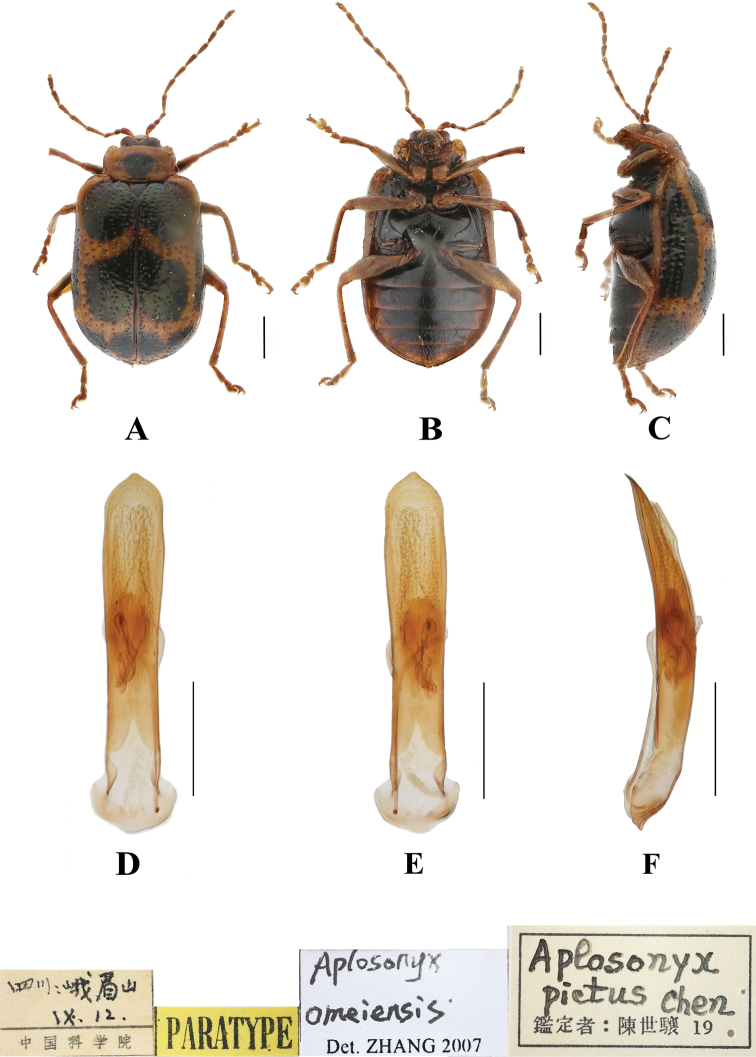
*Aplosonyxomeiensis***A–C** habitus of paratype, IZAS**D–F** aedeagus **A, D** dorsal views **B, E** ventral views **C, F** lateral views. Scale bars 0.5 mm (**D–F**); 1 mm (**A–C**).

Pronotum 1.8 × as wide as long, lateral border margined, widest at anterior 1/3; disc with deep transverse furrow, covered with several punctures in furrow and with sparsely small punctures in other parts of pronotum.

Scutellum triangular, finely covered with punctures.

Elytra wider than pronotum, 0.7 × as long as body, 1.6 × as long as wide, epipleura wide at anterior 1/3, posteriorly gradually narrowing towards apex; dorsal surface slightly convex, covered with punctures in irregular rows, the interstices 2 × as wide as diameter of punctures and slightly covered with fine punctuation.

Metasternum 2 × as long as mesosternum. Ventral surface of abdomen with five ventrites, ventrite 1 longest, ventrites 2–4 gradually shortened, apical ventrite slightly longer than ventrite 3, with two subtriangular incisions.

Aedeagus slender, parallel-sided, slightly narrowed in middle, basally widened, apex pointed, in lateral view slightly bent.

**Female.** Length 4.4–4.8 mm, width 2.6–3.0 mm.

Antennae slightly thinner than in male, antennomere 2 shortest, antennomere 3 approximately 1.5 × as long as second; antennomere 4 longest, 1.5 × as long as antennomeres 2 and 3 combined; apical sternite without incisions.

##### Distribution.

China: Sichuan.

#### ﻿ Aplosonyx orientalis

Taxon classificationAnimaliaColeopteraChrysomelidae

Jacoby, 1892

288A3245-F373-5891-AF28-2078B4F4C20F

**Figure 15.**
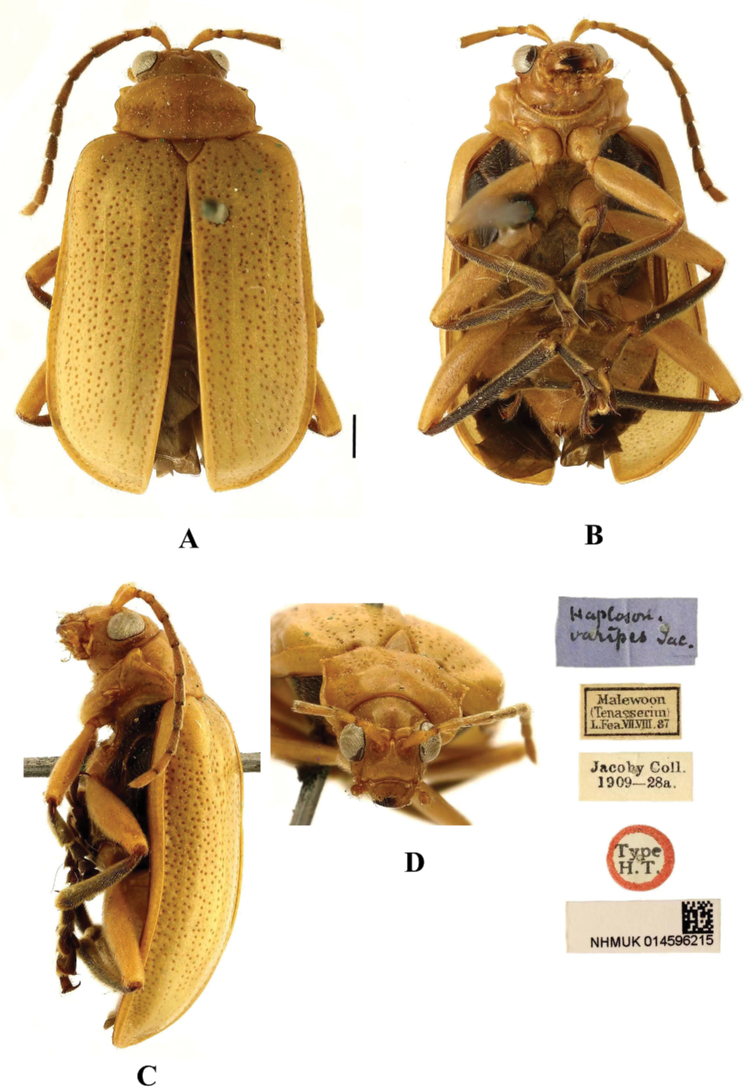
*Aplosonyxorientalis***A–D** habitus of syntype, NHMUK**A** dorsal view **B** ventral view **C** lateral view **D** head view. Scale: 1 mm (**A–D**).

**Figure 16.**
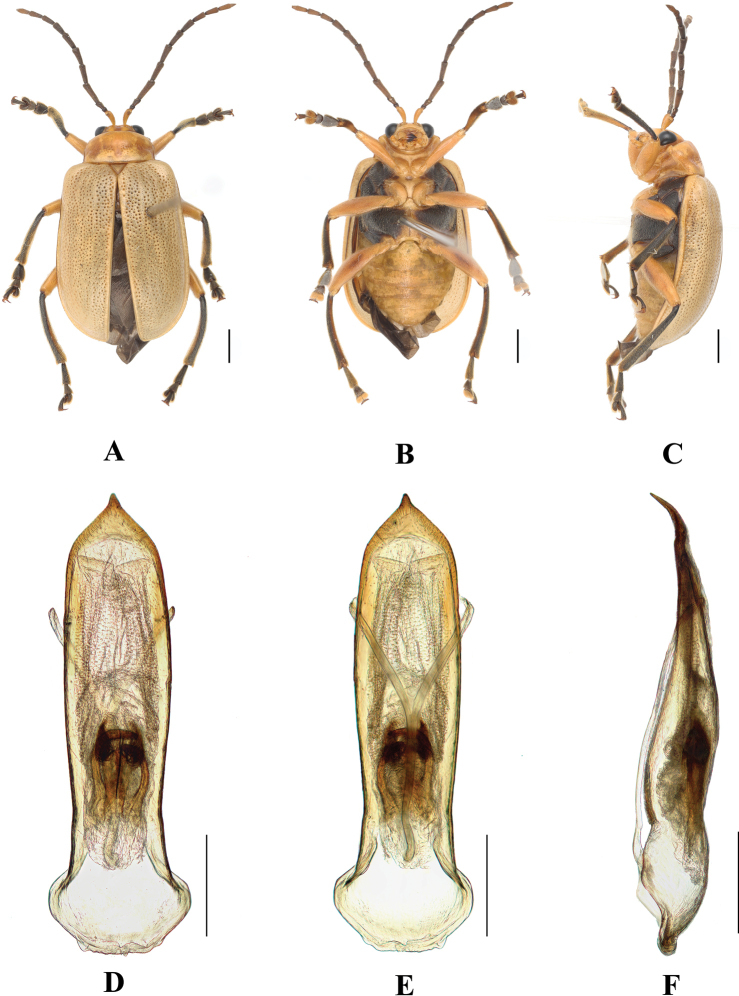
*Aplosonyxorientalis***A–C** habitus **D–F** aedeagus **A, D** dorsal views **B, E** ventral views **C, F** lateral views. Scale bars 0.5 mm (**D–F**); 1 mm (**A–C**).

Figs 11E , 15A–D , 16A–F

Haplosonyx orientalis Jacoby, 1892: 962.
Haplosonyx varipes Jacoby, 1892: 964. Synonymized by Kimoto 1989: 171.
Sphenoraia tonkinensis Laboissière, 1922: 102. Synonymized by Kimoto 1989: 172.
Aplosonyx orientalis : Maulik 1936: 619.

##### Type specimen examined.

♀ ***Syntype*** of *Haplosonyxvaripes*: *Haplosonyxvaripes* Jac.; Malewoon (Tenasserim)L. Fea. VII. VIII. 87; Jacoby Coll. 1909-28a.; Type H. T.; NHMUK 014596215.

##### Additional specimens examined.

8♂♂7♀♀, China, **Guangdong Province**, Enping Qixingkeng; 22 Jun. 2022; Chuan Feng leg.; IZGAS. 4♂♂6♀♀, China, **Guangxi Province**, Maoershan; 25 Aug. 2020; IZGAS. ♂, China, **Yunnan Province**, Xishuangbanna, Menghun; 1200 m a. s. l.; 24 May 1958; Xuwu Meng leg.; IZAS; IOZ(E)1566693. ♂, China, **Yunnan Province**, Xishuangbanna, Menghun; 1200 m a. s. l.; 2 Jun. 1958; Xuwu Meng leg.; IZAS; IOZ(E)1566699. ♂, China, **Yunnan Province**, Xishuangbanna, Menga; 1000 m a. s. l.; 20 May 1958; Fuji Pu leg.; IZAS; IOZ(E)1566695. ♀, China, **Yunnan Province**, Xishuangbanna, Mengzhe; 870 m a. s. l.; 5 Sep. 1958; Shuyong Wang leg.; IZAS; IOZ(E)1566694. ♀, China, **Yunnan Province**, Xishuangbanna, Mengzhe; 870 m a. s. l.; 6 Jul. 1958; Fuji Pu leg.; IZAS; IOZ(E)1566697. ♂, China, **Yunnan Province**, Xishuangbanna, Xiaomengyang; 850 m a. s. l.; 5 Jul. 1958; Lingchao Zang leg.; IZAS; IOZ(E)1566696. ♂, China, **Yunnan Province**, Xishuangbanna, Damenglong; 650 m a. s. l.; 6 May 1958; Chunpei Hong leg.; IZAS; IOZ(E)1566698. ♀, China, **Yunnan Province**, Cheli, Damenglong; 600 m a. s. l.; 29 Apr. 1957; Dahua Liu leg.; IZAS; IOZ(E)1566700.

##### Diagnosis.

This species can be distinguished from other species by the antennae with first antennomere yellow, and antennomeres 2–11 black; the legs are black with yellow femurs. This species differs from *A.cinctus* in having the aedeagus widened towards middle, in lateral view moderately bent.

##### Redescription.

**Male.** Length 9.5–10.4 mm, width 5.0–5.8 mm.

Head, pronotum, elytra, scutellum, and abdomen yellow; antennae black with first antennomere yellow; ventral surface of thorax black and in middle area yellow; legs black with femur, inner sides of tibiae, coxae and trochanters are yellow.

Vertex finely covered with punctures. Interocular space 2 × as wide as transverse diameter of eye. Interantennal space 1.5 × as wide as transverse diameter of antennal socket. Frontal tubercles transverse, each separated by a deep furrow; antennae slender, 0.75 × as long as body; antennomeres 1–3 shiny; antennomeres 4–11 covered with pubescence, antennomere 2 shortest, antennomere 3 approximately 1.8 × as long as second; antennomere 4 longest, approximately 1.4 × as long as antennomeres 2 and 3 combined; antennomeres 5–10 gradually shortened, shorter than antennomere 4; antennomere 11 slightly longer than antennomere 10, pointed.

Pronotum approximately 2 × as wide as long, lateral border margined, widest at posterior corners; disc with deep transverse furrow, closely covered with large punctures in furrow and with sparse small punctures in other parts of pronotum, in furrow the interstices between the punctures equal to diameter of individual punctures.

Scutellum triangular, finely covered with punctures.

Elytra: wider than pronotum, 0.75 × as long as body, 1.55 × as long as wide, epipleura wide at anterior 1/4, posteriorly gradually narrowing towards apex, dorsal surface slightly convex, regularly covered with large and deep punctures, partially arranged in twenty rows in each elytron, the interstices of the punctures in rows approximately 2 × as wide as diameter of punctures and slightly covered with fine punctuation.

Metasternum 2 × as long as mesosternum. Ventral surface of abdomen with five ventrites, ventrite 1 longest, ventrites 2–4 gradually shortened, apical ventrite slightly longer than ventrite 3, with two subtriangular incisions.

Aedeagus gradually widening from base to middle, gradually narrowing from middle towards apex, at one tenth from apex strongly narrowing, ending in a pointed apex. in lateral view moderately bent, with distinctly bent apex.

**Female.** Length 9.2–10.5 mm, width 4.8–5.6 mm.

Antennae slightly thinner than in male, antennomere 2 shortest, antennomere 3 longer than antennomere 2, twice as long as second; antennomere 4 longest, 1.6 × as long as antennomeres 2 and 3 combined; apical sternite without incisions.

##### Distribution.

China: Guangdong, Guangxi, Yunnan; Vietnam, Laos, Thailand, Myanmar, India.

#### ﻿ Aplosonyx ornatus

Taxon classificationAnimaliaColeopteraChrysomelidae

Jacoby, 1892

9358307C-3DB0-57E1-A79A-554E2A3578CC

**Figure 17.**
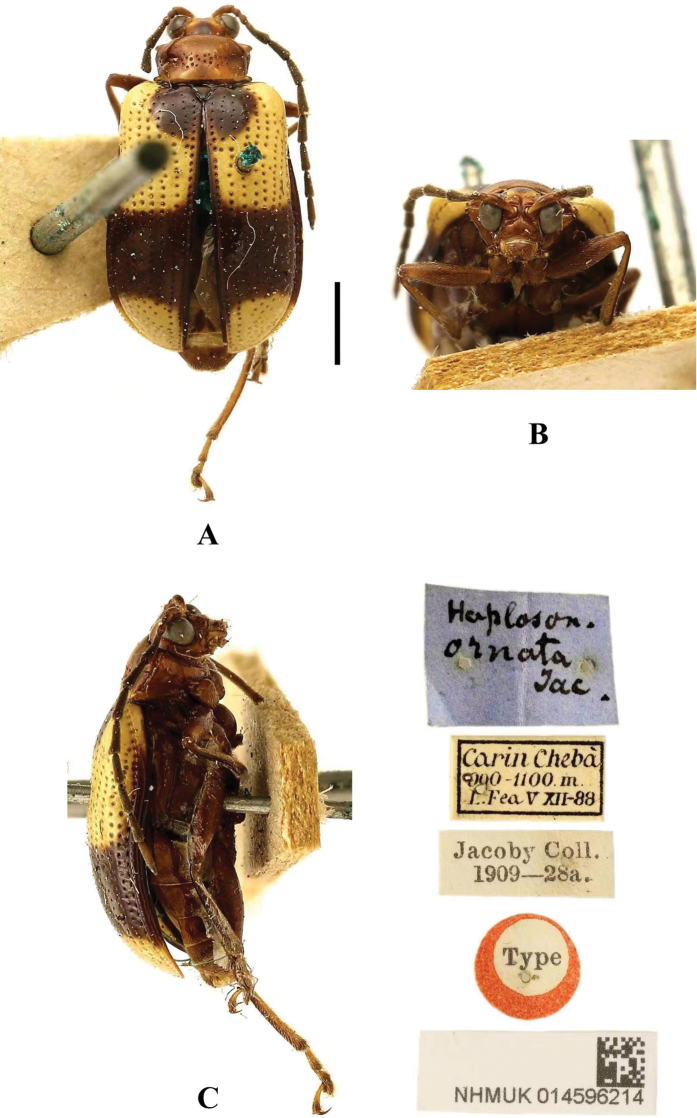
*Aplosonyxornatus***A–C** habitus of syntype, NHMUK014596214 **A** dorsal view **B** head view **C** lateral view. Scale bar: 1 mm (**A–C**).

**Figure 18.**
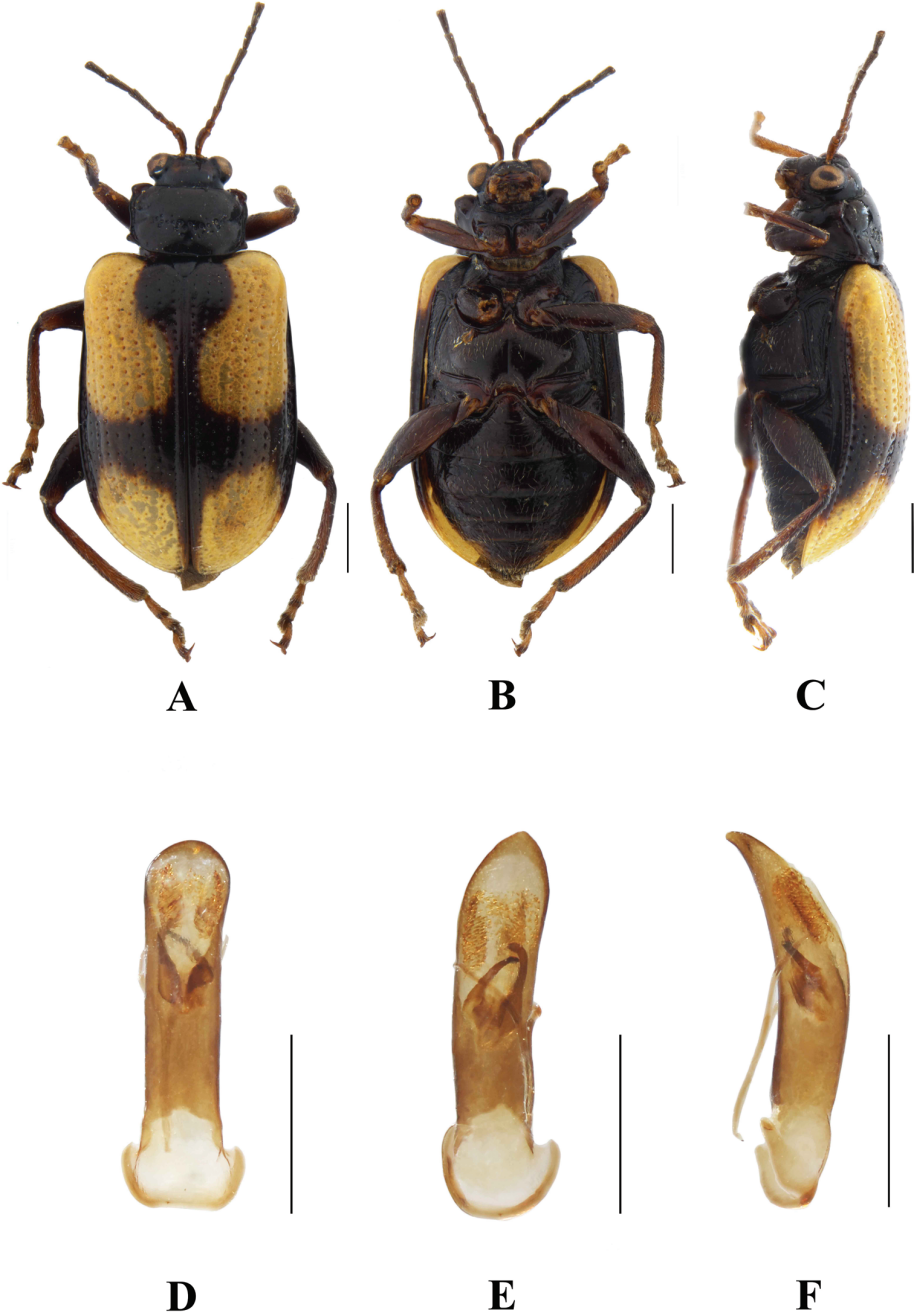
*Aplosonyxornatus***A–C** habitus **D–F** aedeagus **A, D** dorsal views **B, E** ventral views **C, F** lateral views. Scale bars 0.5 mm (**D–F**); 1 mm (**A–C**).

Figs 11F , 17A–C , 18A–F

Haplosonyx ornata Jacoby, 1892: 963.
Aplosonyx ornata : Maulik, 1936: 622.

##### Type specimen examined.

♂ *Haploson.ornata* Jac.; Carin Chebà, 900–1100 m, L. Fea V XII-88; Jacoby Coll., 1909-28a.; syntype, NHMUK014596214.

##### Additional specimens examined.

♀, China, **Yunnan Province**, Xishuangbanna, Menga; 1050 m a. s. l.; 17 May 1958; Shuyong Wang leg.; IZAS; IOZ(E)1566589. ♀, China, **Yunnan Province**, Xishuangbanna, Menga; 1050 m a. s. l.; 12 May 1958; Shuyong Wang leg.; IZAS; IOZ(E)1566595. ♂, China, **Yunnan Province**, Xishuangbanna, Menga; 1080 m a. s. l.; 2 Jun. 1958; Fuji Pu leg.; IZAS; IOZ(E)1566596. ♂, China, **Yunnan Province**, Xishuangbanna, Menga; 1050 m a. s. l.; 2 Jun. 1958; Fuji Pu leg.; IZAS; IOZ(E)1566597. ♂, China, **Yunnan Province**, Xishuangbanna, Menga; 1050 m a. s. l.; 2 Jun. 1958; Fuji Pu leg.; IZAS; IOZ(E)1566596. ♂, China, **Yunnan Province**, Xishuangbanna, Menga; 1080 m a. s. l.; 2 Jun. 1958; Fuji Pu leg.; IZAS; IOZ(E)1566601. ♀, China, **Yunnan Province**, Xishuangbanna, Menghai, nuoshan; 1600 m a. s. l.; 24 Jul. 1958; Shuyong Wang leg.; IZAS; IOZ(E)1566613. 1♂1♀, China, **Yunnan Province**, Xishuangbanna, Menghai, nuoshan; 1200 m a. s. l.; 24 Apr. 1957; Lingchao Zang leg.; IZAS; IOZ(E)1566598. 1♂1♀, China, **Yunnan Province**, Xishuangbanna, Menghai, nuoshan; 1200 m a. s. l.; 28 Apr. 1957; Lingchao Zang leg.; IZAS; IOZ(E)1566600. 2♀♀, China, **Yunnan Province**, Xishuangbanna, Menghai, nuoshan; 1200 m a. s. l.; 24 Apr. 1957; Lingchao Zang leg.; IZAS; IOZ(E)1566617. ♀, China, **Yunnan Province**, Xishuangbanna, Menghun; 1400 m a. s. l.; 17 May 1958; Chunpei Hong leg.; IZAS; IOZ(E)1566590. ♂, China, **Yunnan Province**, Xishuangbanna, Menghun; 1200 m a. s. l.; 24 May 1958; Chunpei Hong leg.; IZAS; IOZ(E)1566591. ♂, China, **Yunnan Province**, Xishuangbanna, Menghun; 1200 m–1400 m a. s. l.; 19 May 1958; Yiran Zhang leg.; IZAS; IOZ(E)1566593. ♀, China, **Yunnan Province**, Xishuangbanna, Menghun; 1200 m a. s. l.; 21 May 1958; Xuwu Meng leg.; IZAS; IOZ(E)1566614. ♀, China, **Yunnan Province**, kunluo; 1050 m a. s. l.; 26 Apr. 1957; Qiuzhen Liang leg.; IZAS; IOZ(E)1566610.

##### Diagnosis.

This species can be distinguished from the other species by its black pronotum and yellow elytra with a broad blackish band in the middle, which extends along the suture and onto the base.

##### Redescription.

**Male.** Length 4.6–5.4 mm, width 3.0–3.4 mm.

Head, antennae, pronotum, scutellum ventral surface of body black or brown; elytra yellow with a broad blackish band in middle, which extends along suture and expends on base.

Vertex finely covered with punctures. Interocular space 1.6 × as wide as transverse diameter of eye. Interantennal space 1.2 × as wide as transverse diameter of antennal socket. Frontal tubercles distinctly raised, hook-like, each separated by a deep furrow; antennae slender, 0.75 × as long as body; antennomeres 1–3 shiny; antennomeres 4–11 covered with pubescence, antennomere 2 shortest, antennomere 3 approximately 1.6 × as long as second; antennomere 4 longest, approximately 1.5 × as long as antennomeres 2 and 3 combined; antennomeres 5–10 gradually shortened, shorter than antennomere 4; antennomere 11 slightly longer than antennomere 10, pointed.

Pronotum approximately 1.5 × as wide as long, lateral border margined, widest at posterior corners; disc with deep transverse furrow, sparsely covered with large punctures in furrow.

Scutellum triangular, finely covered with punctures.

Elytra wider than pronotum, 0.75 × as long as body, 1.8 × as long as wide, epipleura wide at anterior 1/3, posteriorly gradually narrowing towards apex, dorsal surface slightly convex, regularly covered with large and deep punctures, partially arranged in ten rows in each elytron, the interstices between punctures wider than diameter of individual punctures, 2 × as wide as the diameter of individual punctures and lightly covered with small punctures in interstices.

Metasternum 2 × as long as the mesosternum. Ventral surface of abdomen with five ventrites, ventrite 1 longest, ventrites 2–4 gradually shortened, apical ventrite slightly longer than ventrite 3, with two subtriangular incisions.

Aedeagus slender, parallel-sided, basally widened, middle slightly narrowed, apex pointed, in lateral view obviously bent.

**Female.** Length 4.8–5.5 mm, width 2.8–3.2 mm.

Antennae slightly thinner than in male, antennomere 2 shortest, antennomere 3 longer than antennomere 2, twice as long as second; antennomere 4 longest, 1.6 × as long as antennomeres 2 and 3 combined; apical sternite without incisions.

##### Distribution.

China: Yunnan; Laos, Myanmar.

#### ﻿ Aplosonyx pictus

Taxon classificationAnimaliaColeopteraChrysomelidae

Chen, 1942

974CEAC5-BB18-5381-AC72-382B506A36A8

Figs 19A–F , 20A

Aplosonyx pictus Chen, 1942: 39.
Sphenoraia picta : Lopatin 2002: 880.
Aplosonyx pictus : Zhang et al. 2008: 65.

##### Type specimens examined.

***Holotype***: ♂, China, **Gansu Province**; 8 May 1919; IZAS; IOZ(E)215630.

***Paratype***: ♂, China, **Gansu Province**; 8 May 1919; IZAS; IOZ(E)215633. ♀, China, **Gansu Province**; 8 May 1919; IZAS; IOZ(E)215634.

##### Additional specimens examined.

♂, China, **Gansu Province**, Qinghe; 1400 m a. s. l.; 7 Jul. 1999; Jian Yao leg.; IZAS; IOZ(E)1566618. ♂, same data as for preceding; 14 Jul. 1999; Decheng Yuan leg.; IZAS. ♂, same data as for preceding; 14 Jul. 1999; Shuyong Wang leg.; IZAS; IOZ(E)1566619. ♀, same data as for preceding; IOZ(E)1566620. ♂, China, **Shannxi Province**, Taibai Mt; 1850 m a. s. l.; 30 May 1981; IOZ(E)1566622. ♂, same data as for preceding; IOZ(E)1566623. ♀, **Shannxi Province**, Taibai Mt, Haopingsi; 18 Jun. 1981; Xuhui Chai leg.; IOZ(E)1566624.

##### Diagnosis.

This species can be distinguished from other species by each elytron with two longitudinal black stripes, and the apex with one black spot. This species differs from *A.tianpingshanensis* in the aedeagus apex being distinctly pointed; in lateral view the apex is moderately bent.

##### Redescription.

**Male.** Length 4.6–4.9 mm, width 2.5–3.0 mm.

Head, antennae, pronotum, elytra and leg yellow, vertex, scutellum, and ventral surface of the body black, pronotum with a black spot in middle, each elytron with two longitudinal black stripes, and apex with one black spot.

Vertex finely and sparsely covered with punctures. Interocular space 1.5 × as wide as transverse diameter of eye. Interantennal space 1.2 × as wide as transverse diameter of antennal socket. Frontal tubercles transverse, each separated by a deep furrow; antennae slender, 0.7 × as long as body; antennomeres 1–3 shiny; antennomeres 4–11 covered with pubescence, antennomeres 2 and 3 shortest, antennomere 3 nearly equal in length and shape to antennomere 2, antennomere 4 longest, approximately 1.8 × as long as antennomeres 2 and 3 combined; antennomeres 5–10 gradually shortened, shorter than antennomere 4; antennomere 11 slightly longer than antennomere 10, pointed.

**Figure 19.**
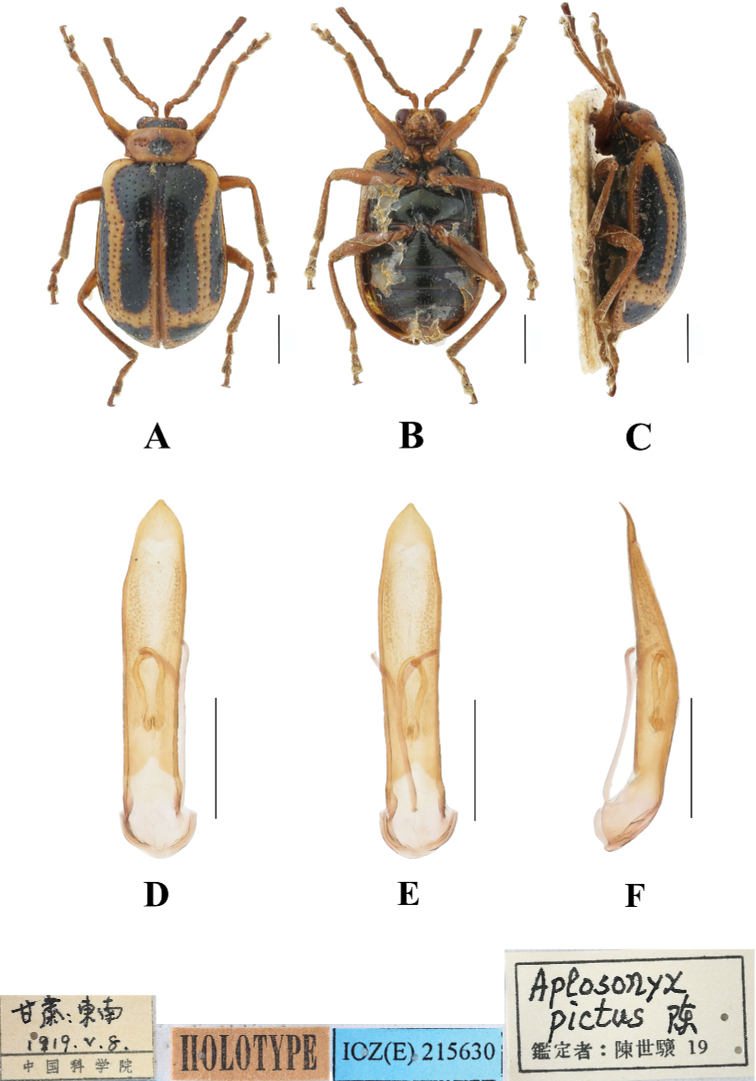
*Aplosonyxpictus***A–C** habitus of holotype, IZAS IOZ(E)215630 **D–F** aedeagus **A, D** dorsal views **B, E** ventral views **C, F** lateral views. Scale bars 0.5 mm (**D–F**); 1 mm (**A–C**).

**Figure 20.**
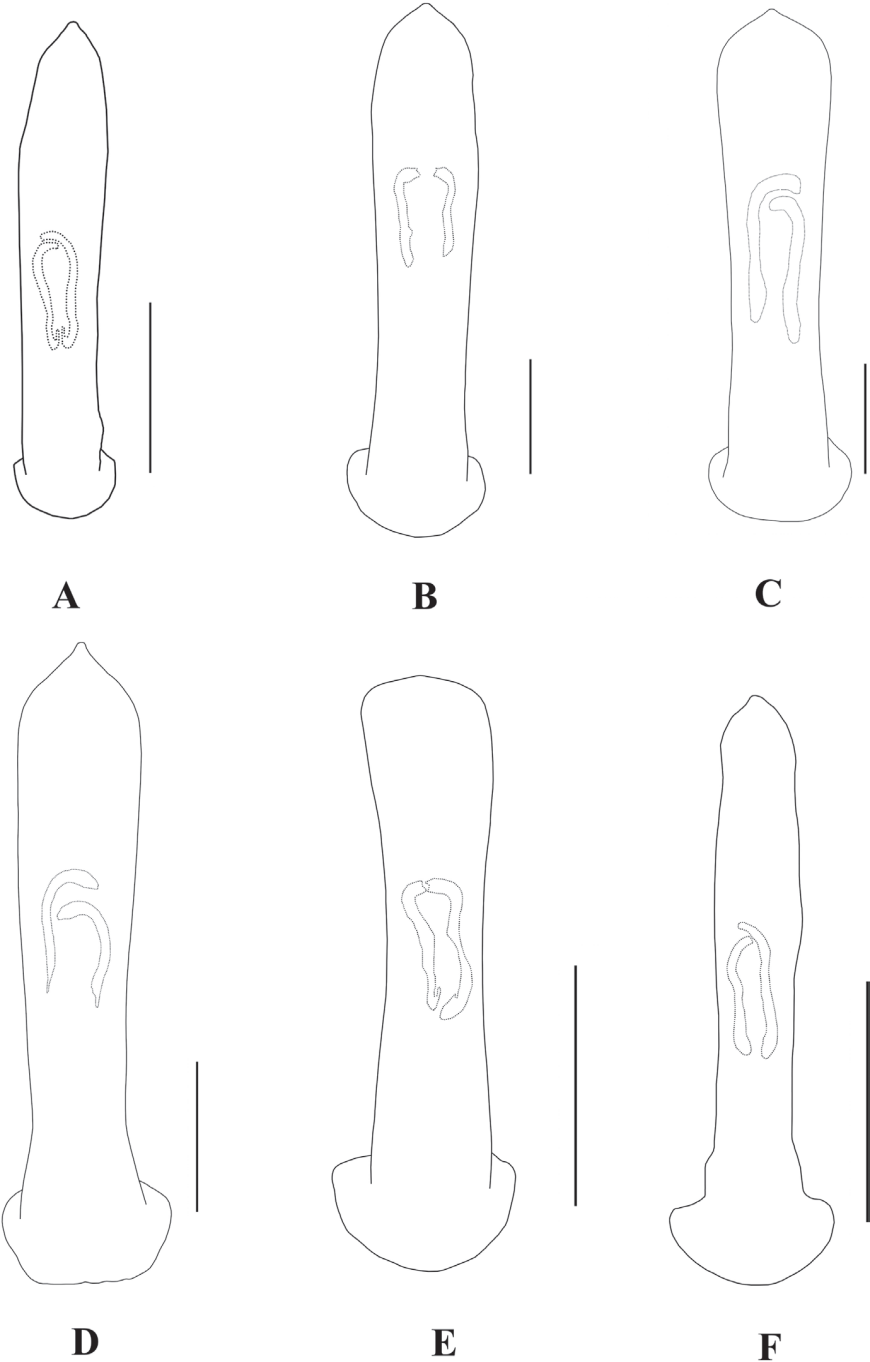
Aedeagus (dorsal view) **A***A.pictus***B***A.robinsoni***C***A.rufipennis***D***A.sublaevicollis***F***A.tianpingshanensis***E***A.yunlongensis*. Scale bars 0.5 mm (**A–F**).

Pronotum 1.8 × as wide as long, lateral border margined, widest at posterior corners; disc with deep transverse furrow, covered with closely large punctures in furrow and with sparsely small punctures in other parts of pronotum.

Scutellum triangular, with rounded apex, smooth, impunctate.

Elytra: wider than pronotum, 0.75 × as long as body, 1.65 × as long as wide, epipleura wide at anterior 1/3, posteriorly gradually narrowing towards apex, dorsal surface slightly convex, regularly covered with large and deep punctures, partially arranged in ten rows in each elytron, the interstices of punctures in rows wider than diameter of punctures, approximately 2 × as wide as diameter of punctures and lightly covered with small punctures in interstices.

Metasternum 2 × as long as the mesosternum. Ventral surface of abdomen with five ventrites, ventrite 1 longest, ventrites 2–4 gradually shortened, apical ventrite slightly longer than ventrite 4, with two subtriangular incisions.

Aedeagus slender, parallel-sided, basally widened, apex distinctly pointed, in lateral view apex moderately bent.

**Female.** Length 4.4–5.0 mm, width 2.6–3.2 mm.

Antennae slightly thinner than in male, without short hairs, antennomere 2 shortest, antennomere 3 approximately 1.5 × as long as second; antennomere 4 longest, approximately 1.5 × as long as antennomeres 2 and 3 combined; apical sternite without incisions.

##### Distribution.

China: Gansu, Shaanxi.

#### ﻿ Aplosonyx robinsoni

Taxon classificationAnimaliaColeopteraChrysomelidae

Jacoby, 1905

A156920C-49F9-5C36-BB85-233CBD76C739

**Figure 21.**
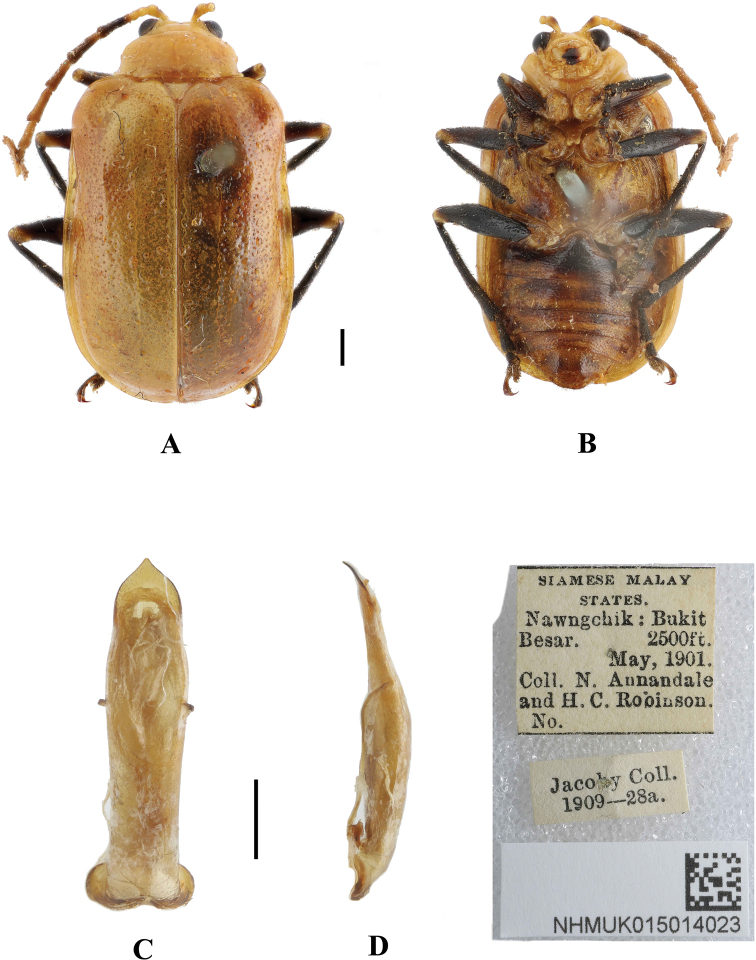
*Aplosonyxrobinsoni***A, B** habitus of syntype, NHMUK015014023 **C, D** aedeagus **A, C** dorsal views **B** ventral views **D** lateral views. Scale bars 0.5 mm (**C, D**); 1 mm (**A, B**).

**Figure 22.**
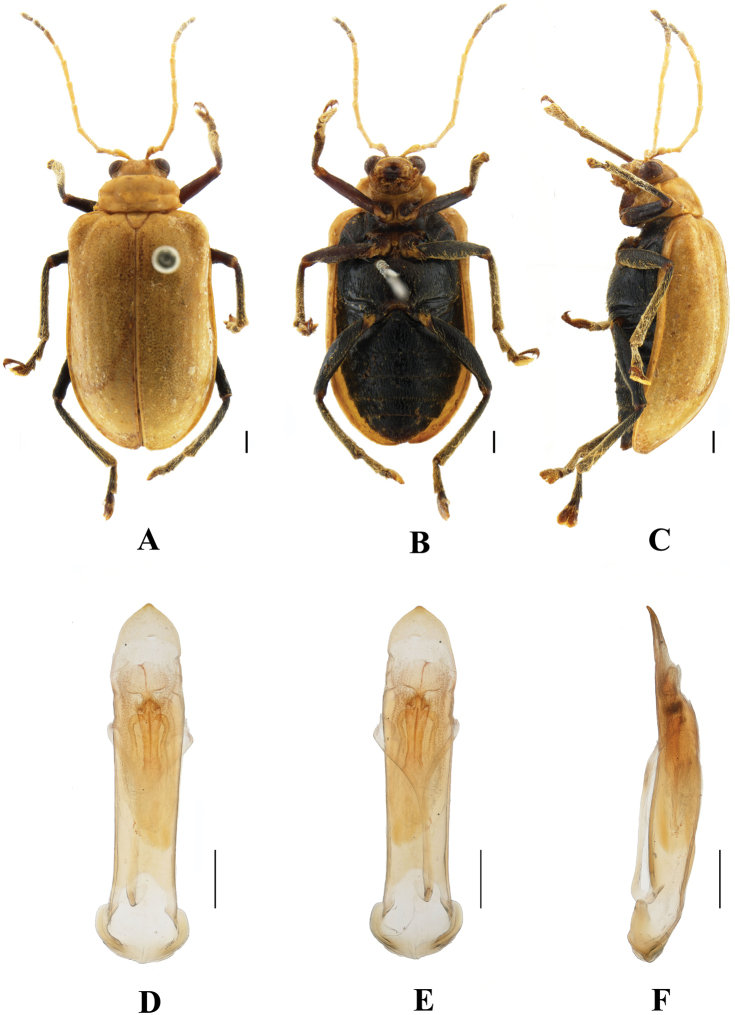
*Aplosonyxrobinsoni***A–C** habitus **D–F** aedeagus **A, D** dorsal views **B, E** ventral views **C, F** lateral views. Scale bars 0.5 mm (**D–F**); 1 mm (**A–C**).

Figs 20B , 21A–D , 22A–F

Haplosonyx robinsoni Jacoby, 1905: 6.
Aplosonyx robinsoni : Maulik 1936: 618.

##### Type specimen examined.

♂ ***Syntype*** of *Haplosonyxrobinsoni* Siamese Malay States. Nawngchik: Bukit Besar. 2500 ft. May 1901. Coll. N. Annandale and H. C. Robinson. No; Jacoby Coll. 1909-28a. NHMUK015014023.

##### Additional specimens examined.

♀, China, **Yunnan Province**, Xishuangbanna, Mengzhe; 870 m a. s. l.; 11 Jul. 1958; Shuyong Wang leg.; IZAS; IOZ(E)1566661. ♀, same data as for preceding; 7 Jul. 1958; Shuyong Wang leg.; IZAS; IOZ(E)1566654. ♂, same data as for preceding; 7 Jul. 1958; Fuji Pu leg.; IZAS; IOZ(E)1566655. ♀, same data as for preceding; IOZ(E)1566657. ♂, China, **Yunnan Province**, Xishuangbanna, Mengzhe; 870 m a. s. l.; 7 Jul. 1958; Fuji Pu leg.; IZAS; IOZ(E)1566660. ♂, same data as for preceding; IOZ(E)1566663. ♀, China, **Yunnan Province**, Xishuangbanna, Mengzhe; 870 m a. s. l.; 7 Jul. 1958; Fuji Pu leg.; IZAS; IOZ(E)1566666. ♀, same data as for preceding; 5 Sep. 1958; Shuyong Wang leg.; IZAS; IOZ(E)1566667. ♀, same data as for preceding; 4 Jul. 1958; Shuyong Wang leg.; IZAS; IOZ(E)1566668. ♀, same data as for preceding; 11 Jul. 1958; Fuji Pu leg.; IZAS; IOZ(E)1566669. ♀, same data as for preceding; IOZ(E)1566670. ♀, China, **Yunnan Province**, Xishuangbanna, Mengzhe; 870 m a. s. l.; 8 Jul. 1958; Shuyong Wang leg.; IZAS; IOZ(E)1566672. ♂, same data as for preceding; 30 Jun. 1958; Shuyong Wang leg.; IZAS; IOZ(E)1566677. ♀, same data as for preceding; 3 Jul. 1958; Shuyong Wang leg.; IZAS; IOZ(E)1566681. ♂, same data as for preceding; 28 Jun. 1957; huyong Wang leg.; IZAS; IOZ(E)1566689. ♂, China, **Yunnan Province**, Jinping; 500 m a. s. l.; 12 May 1956; Keren Huang leg.; IZAS; IOZ(E)1566690. ♀, same data as for preceding; IOZ(E)1566691.

##### Diagnosis.

This species can be distinguished from other species by the yellow body, black legs, and yellow antennae with the apical two or three antennomeres black.

##### Redescription.

**Male.** Length 9.8–13.0 mm, width 6.5–7.4 mm.

Head, pronotum, scutellum, and elytra yellow; legs and ventral surface of the body black; antennae yellow with apical two or three antennomeres black.

Vertex finely covered with punctures. Interocular space 1.5 × as wide as transverse diameter of eye. Interantennal space 1.7 × as wide as transverse diameter of antennal socket. Frontal tubercles transverse, each separated by a deep furrow; antennae slender, 0.7 × as long as body; antennomeres 1–3 shiny; antennomeres 4–11 covered with pubescence, antennomere 2 shortest, antennomere 3 twice as long as second; antennomere 4 longest, 2 × as long as antennomeres 2 and 3 combined; antennomeres 5–10 gradually shortened, shorter than antennomere 4; antennomere 11 slightly longer than antennomere 10, pointed.

Pronotum approximately 2 × as wide as long, lateral border margined, widest at posterior corners; disc with deep transverse furrow, closely covered with large punctures in furrow and sparsely with small punctures in other parts of pronotum, the interstices of punctures equal to diameter of individual punctures in furrow, smooth and impunctate in middle of furrow.

Scutellum triangular, finely covered with punctures.

Elytra: wider than pronotum, 0.8 × as long as body, 1.7 × as long as wide, epipleura wide at anterior 1/4, posteriorly gradually narrowing towards apex, dorsal surface slightly convex, regularly covered with large and deep punctures, partially arranged in twenty rows in each elytron, the interstices of punctures wider than diameter of individual punctures, approximately 2 × as wide as diameter of punctures and lightly covered with small punctures in interstices.

Metasternum 2 × as long as the mesosternum. Ventral surface of abdomen with five ventrites, ventrite 1 longest, ventrites 2–4 gradually shortened, apical ventrite slightly longer than ventrite 3, with two subtriangular incisions.

Aedeagus slender, parallel-sided, basally widened, apex distinctly pointed, in lateral view base and apex moderately bent.

**Female.** Length 10.2–12.8 mm, width 6.6–7.2 mm.

Antennae slightly thinner than in male, antennomere 2 shortest, antennomere 3 twice as long as second; antennomere 4 longest, 1.6 × as long as antennomeres 2 and 3 combined; apical sternite without incisions.

##### Variability.

The syntype studied has different coloration, antennae with antennomeres 1–3 yellow, ventral surface of the body, coxae, and trochanters yellow.

##### Distribution.

China: Yunnan; Thailand, Myanmar, Malaysia, Indonesia.

#### ﻿ Aplosonyx rufipennis

Taxon classificationAnimaliaColeopteraChrysomelidae

Duvivier, 1892

4338AB33-0DEC-5A5B-B08D-6F3EA8CAB75A

**Figure 23.**
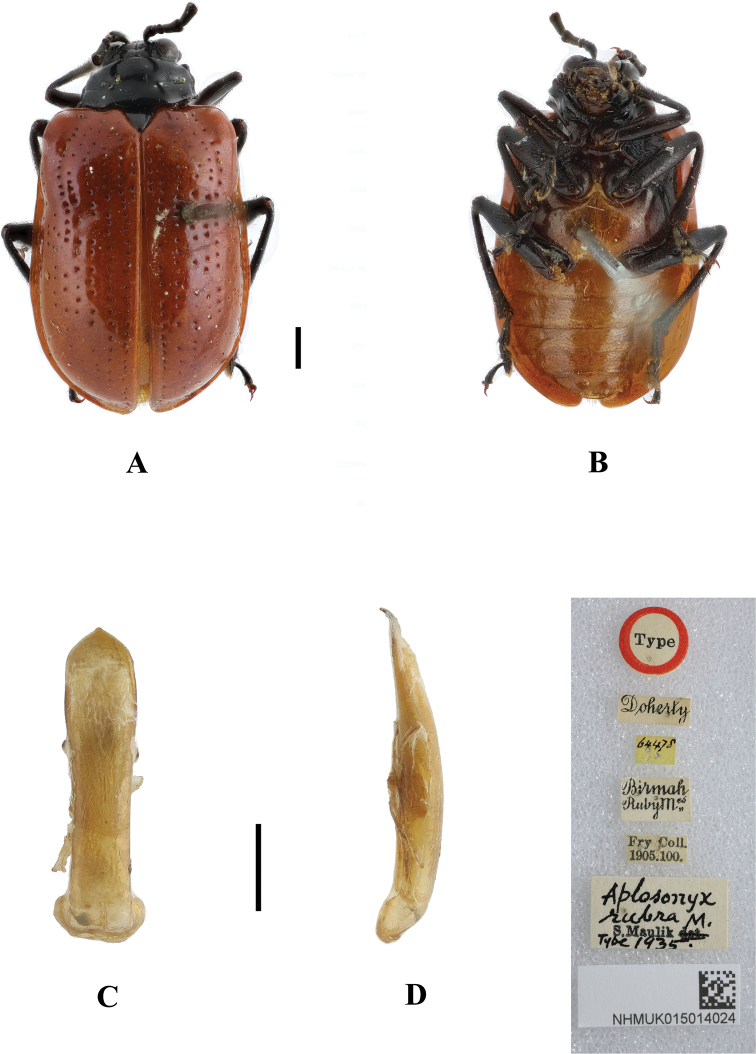
*Aplosonyxrufipennis***A, B** habitus of syntype, NHMUK015014024 **C, D** aedeagus **A, C** dorsal views **B** ventral views **D** lateral views. Scale bars 0.5 mm (**C, D**); 1 mm (**A, B**).

**Figure 24.**
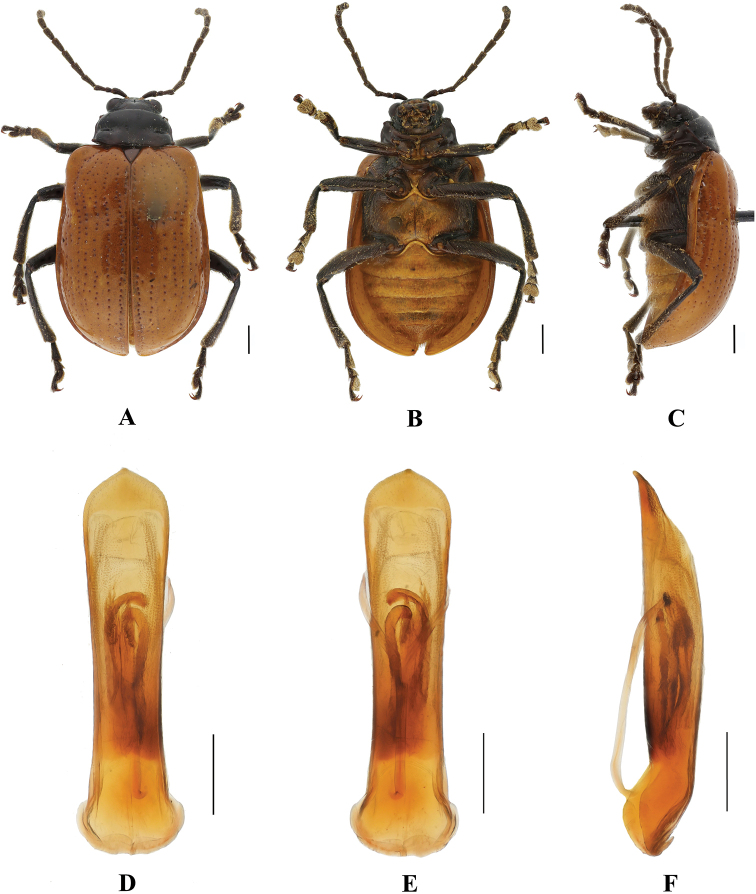
*Aplosonyxrufipennis***A–C** habitus **D–F** aedeagus **A, D** dorsal views **B, E** ventral views **C, F** lateral views. Scale bars 0.5 mm (**D–F**); 1 mm (**A–C**).

Figs 20C , 23A–D , 24A–F

Haplosonyx rufipennis Duvivier, 1892: 439.
Aplosonyx rufipennis : Laboissière 1934: 110.
Aplosonyx rubra Maulik, 1936: 620. Synonymized by Laboissière 1940: 21.

##### Type specimen examined.

♂ ***Syntype*** of *Aplosonyxrubra*: Doherty, 64478, Birmah Ruby M^es^; Fry Coll., 1905. 100; *Aplosonyxrubra* M.; S. Maulik; Type 1935, NHMUK015014024.

##### Additional specimens examined.

♀, China, **Yunnan Province**, Pingbian; 700 m a. s. l.; 29 Jun. 1956; Bangfeiluofu leg.; IZAS; IOZ(E)1566821. ♂, China, **Yunnan Province**, Hekou; 80 m a. s. l.; 5 Jun. 1956; Keren Huang leg.; IZAS; IOZ(E)1566825. ♀, China, **Yunnan Province**, Pingbian; 800 m a. s. l.; 20 Jun. 1979; Baowen Zhang leg.; IZAS; IOZ(E)1566832. ♂, China, **Yunnan Province**; 1956; IOZ(E)1566822. ♀, China, **Yunnan Province**; 1956; IOZ(E)1566823. ♀, same data as for preceding; IOZ(E)1566824.

##### Diagnosis.

This species can be distinguished from other species by its black head and pronotum, and the reddish brown elytra without any spots.

##### Redescription.

**Male.** Length 8.4–10.6 mm, width 5.2–6.0 mm.

Head, antennae, pronotum, scutellum and leg black, elytra reddish brown, ventral surface of thorax yellow with lateral area black, abdomen yellow.

Vertex finely covered with punctures. Interocular space 1.6 × as wide as transverse diameter of eye. Interantennal space 1.5 × as wide as transverse diameter of antennal socket. Frontal tubercles transverse, each separated by a deep furrow; antennae slender, 0.65 × as long as body; antennomeres 1–3 shiny; antennomeres 4–11 covered with pubescence, antennomere 2 shortest, antennomere 3 approximately 1.5 × as long as second; antennomere 4 longest, approximately 1.8 × as long as antennomeres 2 and 3 combined; antennomeres 5–10 gradually shortened, shorter than antennomere 4; antennomere 11 slightly longer than antennomere 10, pointed.

Pronotum 1.8 × as wide as long, lateral border margined, widest at posterior corners; disc with deep transverse furrow, less distinct, smooth and impunctate in middle; covered with several large punctures in furrow and with sparsely small punctures in other parts of pronotum.

Scutellum triangular, finely covered with punctures.

Elytra: wider than pronotum, 0.75 × as long as body, 1.65 × as long as wide, epipleura wide at anterior 1/4, posteriorly gradually narrowing towards apex, dorsal surface convex slightly, covered with large punctures regularly, partially arranged in ten rows in each elytron, the interstices of punctures wider than diameter of punctures, approximately 2 × as wide as diameter of punctures, covered with small punctures.

Metasternum 2 × as long as the mesosternum. Ventral surface of abdomen with five ventrites, ventrite 1 longest, ventrites 2–4 gradually shortened, apical ventrite slightly longer than ventrite 3, with two subtriangular incisions.

Aedeagus slender, parallel-sided, basally widened, narrowed in middle, apex round with slightly pointed, in lateral view base and apex slightly bent.

**Female.** Length 9.2–10.5 mm, width 4.8–5.6 mm.

Antennomere 2 shortest, antennomere 3 longer than antennomere 2, twice as long as second; antennomere 4 longest, slightly long than antennomeres 2 and 3 combined; apical sternite without incisions.

##### Distribution.

China: Shanghai, Yunnan; Vietnam, India.

#### ﻿ Aplosonyx sublaevicollis

Taxon classificationAnimaliaColeopteraChrysomelidae

Jacoby, 1889

6809E4BE-E41F-51B1-93A1-FDF9B592FE27

Figs 20D , 25A–F

Haplosonyx sublaevicollis Jacoby, 1889: 218.
Aplosonyx sublaevicollis : Maulik 1936: 615.

##### Additional specimens examined.

♂, China, **Yunnan Province**, Xishuangbanna, Xiaomengyang; 850 m a. s. l.; 25 Jun. 1957; Lingchao Zang leg.; IZAS; IOZ(E)1566505. ♂, same data as for preceding; 24 Jun. 1957; Lingchao Zang leg.; IZAS; IOZ(E)1566514. ♂, China, **Yunnan Province**, Xishuangbanna, Damenglong; 650 m a. s. l.; 6 May 1958; IZAS; IOZ(E)1566540. ♂, same data as for preceding; IZAS; IOZ(E)1566541. ♂, China, **Yunnan Province**, Xishuangbanna, Damenglong; 650 m a. s. l.; 7 May 1958; Chunpei Hong leg.; IZAS; IOZ(E)1566508. ♂, same data as for preceding; IOZ(E)1566510. ♂, China, **Yunnan Province**, Xishuangbanna, Damenglong; 650 m a. s. l.; 6 May 1958; Zhizi Chen leg.; IZAS; IOZ(E)1566513. ♂, same data as for preceding; Chunpei Hong leg.; IZAS; IOZ(E)1566517. ♂, China, **Yunnan Province**, Xishuangbanna, Damenglong; 650 m a. s. l.; 6 May 1958; Shuyong Wang leg.; IZAS; IOZ(E)1566518. ♀, same data as for preceding; 7 Oct. 1958; Zhizi Chen leg.; IZAS; IOZ(E)1566534 ♂, same data as for preceding; 7 Oct. 1958; Zhizi Chen leg.; IZAS; IOZ(E)1566535. ♀, China, **Yunnan Province**, Xishuangbanna, Menghun; 750 m a. s. l.; 2 Jun. 1958; Chunpei Hong leg.; IZAS; IOZ(E)1566507. ♂, same data as for preceding; 5 Jun. 1958; Chunpei Hong leg.; IZAS; IOZ(E)1566512. ♂, same data as for preceding; 30 May 1958; IZAS; IOZ(E)1566515. ♂, same data as for preceding; 30 May 1958; IZAS; IOZ(E)1566516. ♂, same data as for preceding; 30 May 1958; Chunpei Hong leg.; IZAS; /IOZ(E)1566519. ♀, China, **Yunnan Province**, Xishuangbanna, Yunjinghong; 900 m a. s. l.; 27 May 1958; Yiran Zhang leg.; IZAS; IOZ(E)1566509. ♂, China, **Yunnan Province**, Xishuangbanna, Mengla; 650 m a. s. l.; 18 May 1958; Fuji Pu leg.; IZAS; IOZ(E)1566520. ♀, China, **Yunnan Province**, Xishuangbanna, Menghun; 1400 m a. s. l.; 3 Jun. 1958; Shuyong Wang leg.; IZAS; IOZ(E)1566556.

##### Diagnosis.

This species can be distinguished from other Chinese species by its purplish blue elytra, and the apex of the pronotum without a raised area. This species differs from *A.chalybeus* in the pronotum being widest at its posterior corners.

##### Redescription.

**Male.** Length 9.0–10.8 mm, width 4.8–5.2 mm.

Head, antennae, pronotum, scutellum and ventral surface of body yellow, legs brown with femur yellow, elytra purplish blue.

Vertex covered with several large punctures. Interocular space 1.65 × as wide as transverse diameter of eye. Interantennal space 1.4 × as wide as transverse diameter of antennal socket. Frontal tubercles distinctly raised, hook-like, each separated by a deep furrow; antennae slender, 0.75 × as long as body; antennomeres 1–3 shiny; antennomeres 4–11 covered with pubescence, antennomere 2 shortest, antennomere 3 twice as long as second; antennomere 4 longest, approximately 1.8 × as long as antennomeres 2 and 3 combined; antennomeres 5–10 gradually shortened, shorter than antennomere 4; antennomere 11 slightly longer than antennomere 10, pointed.

**Figure 25.**
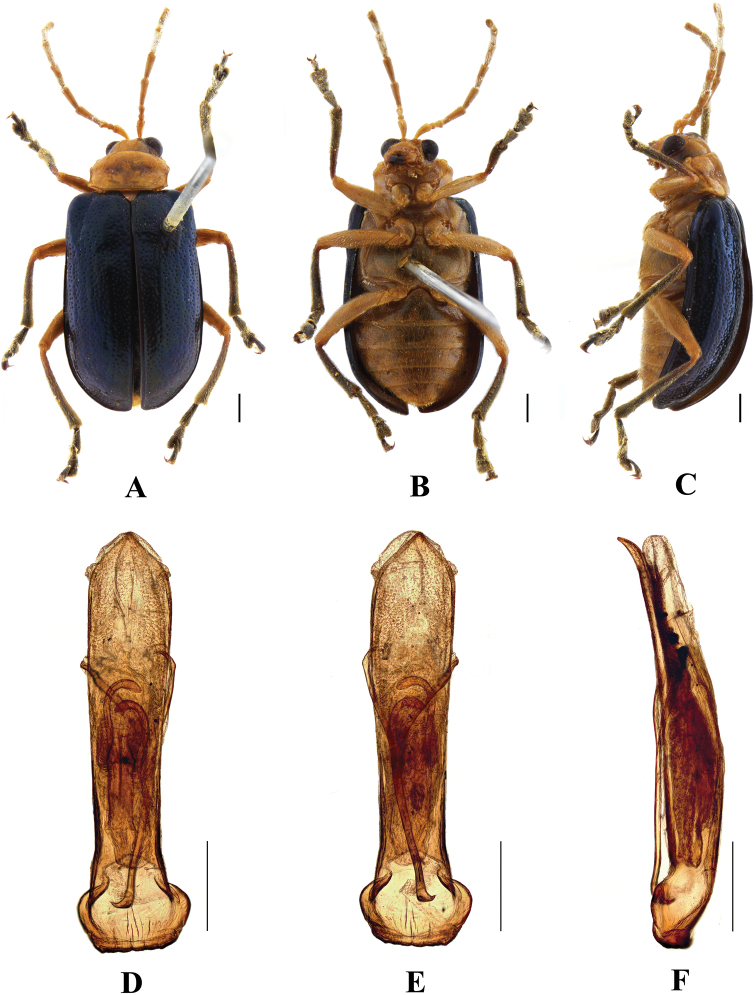
*Aplosonyxsublaevicollis***A–C** habitus **D–F** aedeagus **A, D** dorsal views **B, E** ventral views **C, F** lateral views. Scale bars 0.5 mm (**D–F**); 1 mm (**A–C**).

Pronotum approximately 2 × as wide as long, lateral border margined, widest at posterior corners; middle of disc with transverse furrow; closely covered with large punctures in furrow and with sparsely small punctures in other parts of pronotum.

Scutellum triangular, smooth, impunctate.

Elytra: wider than pronotum, 0.8 × as long as body, 1.7 × as long as wide, epipleura wide at anterior 1/4, posteriorly gradually narrowing towards apex, dorsal surface slightly convex, irregularly covered with large punctures, the interstices of punctures wider than diameter of individual punctures, approximately 2 × as wide as diameter of individual punctures and lightly covered with small punctures in interstices.

Metasternum 2 × as long as the mesosternum. Ventral surface of abdomen with five ventrites, ventrite 1 longest, ventrites 2–4 gradually shortened, apical ventrite slightly longer than ventrite 3, with two subtriangular incisions.

Aedeagus slender, parallel-sided, basally widened, narrowed in middle, apex distinctly pointed, in lateral view base and apex distinctly bent.

**Female.** Length 8.8–10.6 mm, width 4.6–5.4 mm.

Antennae slightly thinner than in male, antennomere 2 shortest, antennomere 3 approximately 1.5 × as long as second; antennomere 4 longest, 1.5 × as long as antennomeres 2 and 3 combined; apical sternite without incisions.

##### Distribution.

China: Yunnan; Laos; Thailand; Myanmar; Malaysia; Indonesia.

#### ﻿ Aplosonyx tianpingshanensis

Taxon classificationAnimaliaColeopteraChrysomelidae

Yang, 1995

5E1E4ACA-D19A-550F-92D3-5DC592A0095F

Figs 20E , 26A–F

Aplosonyx tianpingshanensis Yang, 1995: 91.

##### Type specimens examined.

***Holotype***: ♂, China, **Hunan Province**, Sangzhi, Tianping Mt; 1640 m a. s. l.; 13 Aug. 1988; Xingke Yang leg.; IZAS.

***Paratype***: ♀, same data as for holotype. ♀, China, **Hubei Province**, Hefeng, Fenshuiling; 1250 m a. s. l.; 3 Aug. 1989; Xiaochun Zhang leg.; IZAS. ♂, China, **Hunan Province**, Sangzhi, Tianping Mt; 1570 m a. s. l.; 13 Aug. 1988; Shuyong Wang leg.; IZAS. ♂, same data as for preceding; 1640 m a. s. l.; 13 Aug. 1988; Xingke Yang leg.; IZAS.

##### Additional specimen examined.

♀, China, **Hunan Province**, Sangzhi, Tianping Mt; 1640 m a. s. l.; 14 Aug. 1988; Xingke Yang leg.; IZAS; IOZ(E)1566662.

##### Diagnosis.

This species can be distinguished from the other Chinese species by each elytron having two broad longitudinal black stripes, and the apex with two black spots. This species differs from *A.pictus* in the aedeagus being slightly narrowed in the middle, and the apex widened.

##### Redescription.

**Male.** Length 5.0–5.3 mm, width 3.0–3.2 mm.

Head, antennae, pronotum, elytra and legs yellow, vertex, scutellum and ventral surface of the body black, margin and apex of abdominal ventrite yellow, pronotum with a black spot in middle, each elytron with two longitudinal black stripes, and apex with one pair of black spots.

Vertex finely and sparsely covered with punctures. Interocular space 2 × as wide as transverse diameter of eye. Interantennal space 1.4 × as wide as transverse diameter of antennal socket. Frontal tubercles transverse, each separated by a deep furrow; antennae slender, 0.7 × as long as body; antennomeres 1–3 shiny; antennomeres 4–11 covered with pubescence, antennomere 2 shortest, antennomere 3 approximately 1.5 × as long as second; antennomere 4 longest, approximately 1.6 × as long as antennomeres 2 and 3 combined; antennomeres 5–10 gradually shortened, shorter than antennomere 4; antennomere 11 slightly longer than antennomere 10, pointed.

**Figure 26.**
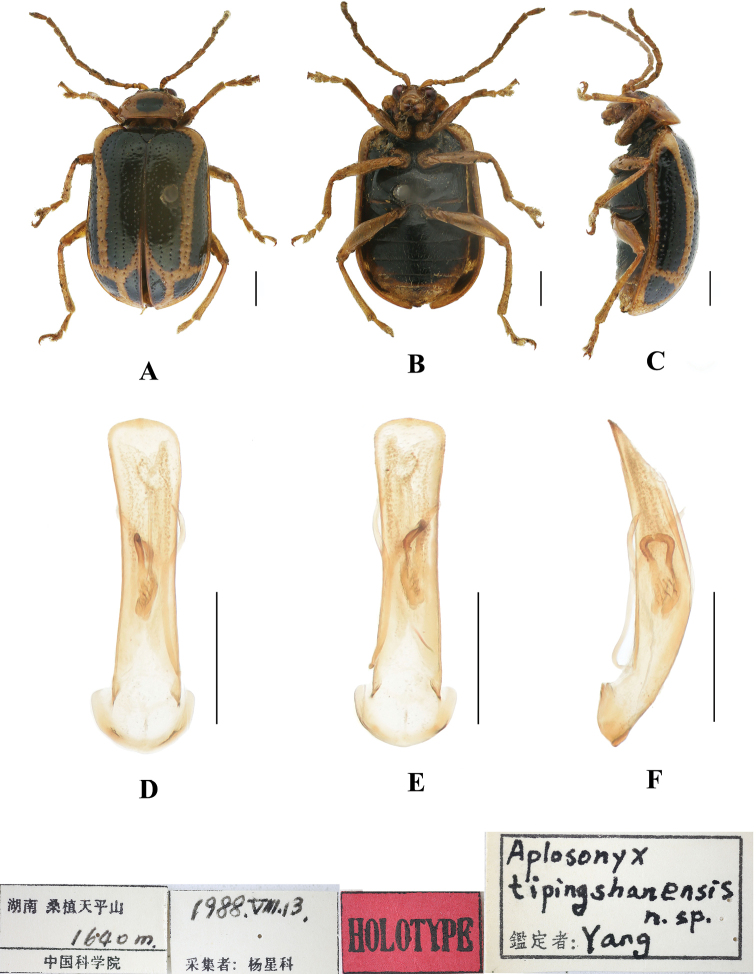
*Aplosonyxtianpingshanensis***A–C** habitus of holotype, IZAS**D–F** aedeagus **A, D** dorsal views **B, E** ventral views **C, F** lateral views. Scale bars 0.5 mm (**D–F**); 1 mm (**A–C**).

Pronotum approximately 1.8 × as wide as long, lateral border margined, widest at posterior corners; disc with deep transverse furrow, covered with several punctures in furrow and with sparsely small punctures in anterior angle.

Scutellum triangular, smooth, impunctate.

Elytra: wider than pronotum, 0.75 × as long as body, 1.75 × as long as wide, epipleura wide at anterior 1/3, posteriorly gradually narrowing towards apex, dorsal surface slightly convex, regularly covered with large and deep punctures, partially arranged in ten rows in each elytron, the interstices of punctures wider than diameter of punctures, approximately 2 × as wide as diameter of punctures and lightly covered with small punctures in interstices.

Metasternum 2 × as long as the mesosternum. Ventral surface of abdomen with five ventrites, ventrite 1 longest, ventrites 2–4 gradually shortened, apical ventrite slightly longer than ventrite 3, with two subtriangular incisions.

Aedeagus slender, parallel-sided, slightly narrowed in middle, basally widened, apex widened, in lateral view strongly bent.

**Female.** Length 5.0–5.2 mm, width 2.8–3.2 mm.

Antennae slightly thinner than in male, antennomere 2 shortest, antennomere 3 approximately 1.6 × as long as second; antennomere 4 longest, 1.4 × as long as antennomeres 2 and 3 combined; punctures densely in groove of pronotum, the interstices between punctures equal to diameter of individual punctures, apical sternite without incisions.

##### Distribution.

China: Gansu, Shaanxi, Hunan, Hubei, Guizhou.

#### ﻿ Aplosonyx yunlongensis

Taxon classificationAnimaliaColeopteraChrysomelidae

Jiang, 1992

30F865A0-0AE8-5BEF-888C-83514D95EC7C

Fig. 27A–F

Aplosonyx yunlongensis Jiang, 1992: 664.

##### Type specimens examined.

***Holotype***: ♂, China, **Yunnan Province**, Yunlong, Zhiben Mt; 2250 m a. s. l.; 21 Jun. 1981; Shuyong Wang leg.; IZAS.

***Paratype***: ♀, China, **Yunnan Province**, Yunlong, Zhiben Mt; 2250 m a. s. l.; 21 Jun. 1981; Shuyong Wang leg.; IZAS.

##### Additional specimen examined.

♀, China, **Yunnan Province**, Yunlong, Zhiben Mt; 2250 m a. s. l.; 21 Jun. 1981; Shuyong Wang leg.; IZAS.

##### Diagnosis.

This species can be distinguished from other Chinese species by each elytron having five black spots, the pronotum with three obvious black spots. This species differs from *A.omeiensis* in the aedeagus being slightly widened at the middle, and the base expanded into a fan shape.

##### Redescription.

**Male.** Length 5.4–5.8 mm, width 3.6–3.8 mm.

Head, antennae, pronotum, elytra, and leg yellow; vertex, scutellum, and ventral surface of the body black, apical ventrite of abdomen yellow, pronotum with three black spots, one large black spot in the middle, and one small black spot on each side; each elytron with five black spots, middle and apex with one pair of spots and base with one spot.

Vertex finely and sparsely covered with punctures. Interocular space 1.4 × as wide as transverse diameter of eye. Interantennal space 1.3 × as wide as transverse diameter of antennal socket. Frontal tubercles distinctly raised, hook-like, each separated by a deep furrow; antennae slender, 0.75 × as long as body; antennomeres 1–3 shiny; antennomeres 4–11 covered with pubescence, antennomere 2 shortest, antennomere 3 twice as long as second; antennomere 4 longest, approximately 1.6 × as long as antennomeres 2 and 3 combined; antennomeres 5–10 gradually shortened, shorter than antennomere 4; antennomere 11 slightly longer than antennomere 10, pointed.

**Figure 27.**
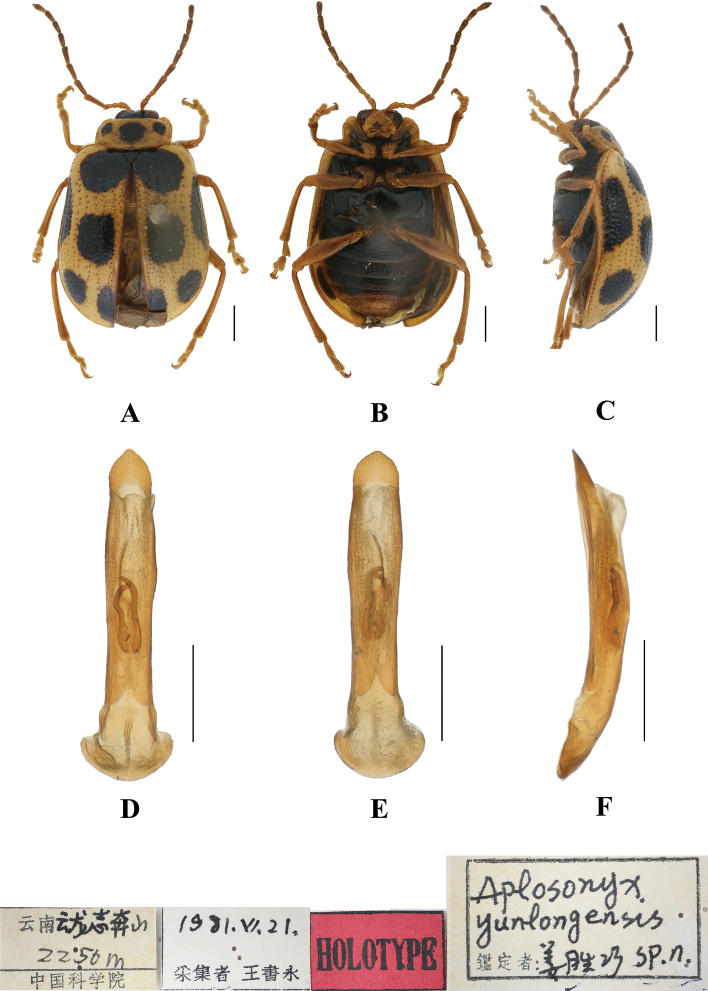
*Aplosonyxyunlongensis***A–C** habitus of holotype, IZAS**D–F** aedeagus **A, D** dorsal views **B, E** ventral views **C, F** lateral views. Scale bars 0.5 mm (**D–F**); 1 mm (**A–C**).

Pronotum approximately 2 × as wide as long, lateral border margined, widest at posterior corner; disc with transverse furrow, less distinct in middle; sparsely covered with several large punctures in furrow.

Scutellum triangular, finely covered with punctures.

Elytra: wider than pronotum, 0.78 × as long as body, 1.7 × as long as wide, epipleura wide at anterior 1/3, posteriorly gradually narrowing towards apex, dorsal surface slightly convex, regularly covered with large and deep punctures, partially arranged in ten rows on each elytron, the interstices of punctures 2 × as wide as diameter of punctures and lightly covered with small punctures.

Metasternum 2 × as long as the mesosternum. Ventral surface of abdomen with five ventrites, ventrite 1 longest, ventrites 2–4 gradually shortened, apical ventrite slightly longer than ventrite 3, with two subtriangular incisions.

Aedeagus slender, slightly widened at middle, basally enlarged in a fan shape, apex pointed, in lateral view slightly bent.

**Female.** Length 5.6–6.0 mm, width 3.5–3.9 mm.

Antennae slightly thinner than in male, antennomere 2 shortest, antennomere 3 approximately 1.5 × as long as second; antennomere 4 longest, 1.3 × as long as antennomeres 2 and 3 combined; apical sternite without incisions.

##### Distribution.

China: Yunnan.

#### ﻿ Aplosonyx ancorella sp. nov.

Taxon classificationAnimaliaColeopteraChrysomelidae

19C16106-2922-5459-907F-7117693EF612

https://zoobank.org/1C9DA9C0-CDCD-44DA-9E5A-B9ADA4B2B32D

Figs 28A–F , 29A

##### Type material.

***Holotype***: ♂, China, **Yunnan Province**, Menga; 1100 m a. s. l.; 18 Apr. 1982; Subai Liao leg.; IZAS; IOZ(E)1566748. ***Paratype***: 1♂, China, **Yunnan Province**, Xiaomengyang; 850 m a. s. l.; 7 May 1957; Fuji Pu leg.; IZAS; IOZ(E)1566747.

##### Diagnosis.

The new species closely resembles *A.ancora* and *A.fulvescens.* In *A.ancora*, the antennae with antennomeres 1–7 yellow and antennomeres 8–11 brown, abdomen with five pairs of black spots, the pronotum and elytra densely covered with large punctures, and the interstices of the punctures in the elytra are somewhat wrinkled. In *A.fulvescens*, the antennae with antennomeres 1–3 yellow and antennomeres 4–11 brown, the pronotum and elytra are sparsely covered with small punctures.

##### Description.

**Male.** Length 10.8–12.0 mm, width 5.8–6.2 mm.

Head, pronotum, abdomen and leg yellow, elytra reddish brown, antennae with antennomeres 1–7 yellow and antennomeres 8–11 brown, scutellum black, ventral surface of thorax black with yellow middle, pronotum purple or black, with lateral margin and anterior margin yellow, elytra with a broad purplish band from anterior to middle, which extends forward along suture and expends again on base, abdomen with five pair of black spots at side on each visible sternites.

Vertex finely and sparsely covered with punctures. Interocular space 1.9 × as wide as transverse diameter of eye. Interantennal space 1.3 × as wide as transverse diameter of antennal socket. Frontal tubercles transverse, each separated by a deep furrow; antennae slender, 0.7 × as long as body; antennomeres 1–3 shiny; antennomeres 4–11 with short hairs, antennomere 2 shortest, antennomere 3 approximately 1.5 × as long as second; antennomere 4 longest, approximately 2 × as long as antennomeres 2 and 3 combined; antennomeres 5–10 gradually shortened, shorter than antennomere 4; antennomere 11 slightly longer than antennomere 10, pointed.

**Figure 28.**
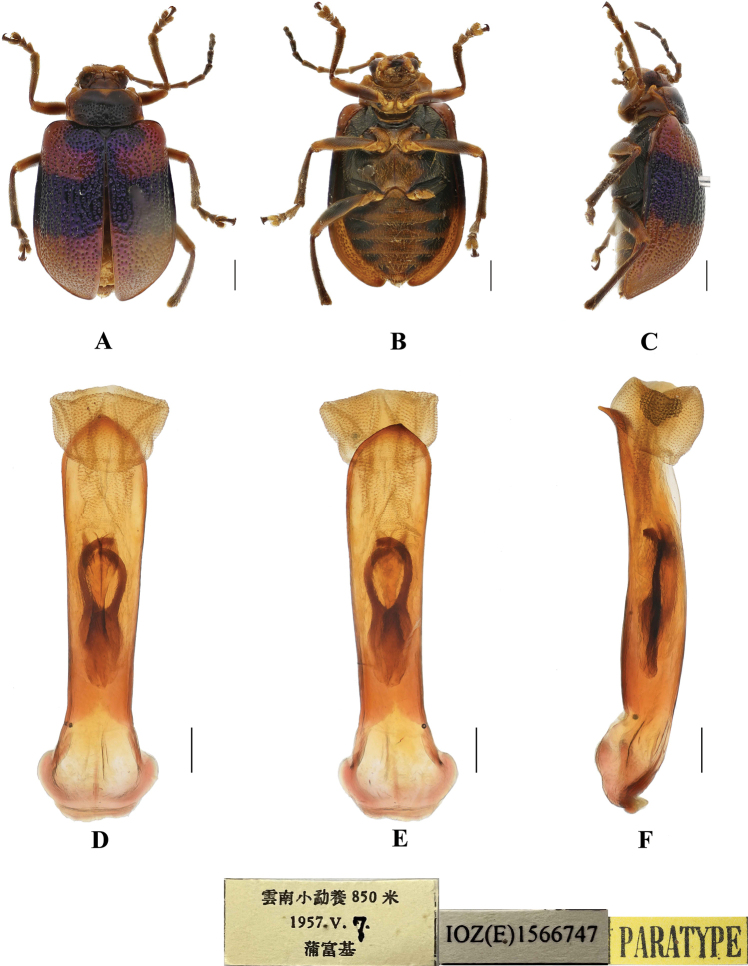
*Aplosonyxancorella* sp. nov. **A–C** habitus of paratype, IZAS**D–F** aedeagus **A, D** dorsal views **B, E** ventral views **C, F** lateral views. Scale bars 0.5 mm (**D–F**); 1 mm (**A–C**).

**Figure 29.**
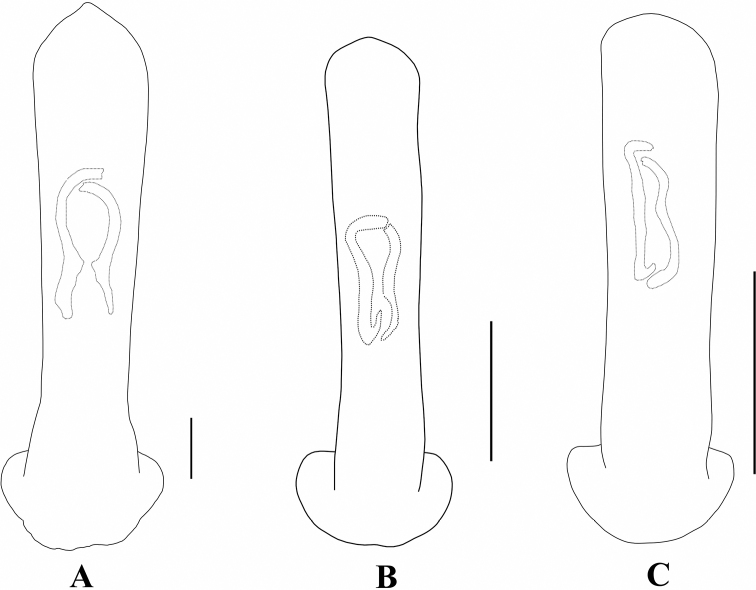
Aedeagus (dorsal view) **A***A.ancorella* sp. nov. **B***A.nigricornis* sp. nov. **C***A.wudangensis* sp. nov. Scale bars: 0.5 mm (**A–F**).

Pronotum approximately 2 × as wide as long, disc with transverse furrow, densely covered with large punctures, the interstices of punctures distinctly narrower than diameter of punctures and with sparsely small punctures in apex of pronotum.

Scutellum triangular, finely covered with punctures.

Elytra: wider than pronotum, 0.7 × as long as body, 1.5 × as long as wide, epipleura wide at anterior 1/4, posteriorly gradually narrowing towards apex, dorsal surface slightly convex, irregularly covered with large and deep punctures, the interstices of punctures narrower than diameter of punctures and lightly covered with small punctures in interstices. their interstices somewhat wrinkled.

Metasternum 2 × as long as mesosternum. Ventral surface of abdomen with five ventrite, ventrite 1 longest, ventrites 2–4 gradually shortened, apical ventrite slightly longer than ventrite 3, with two subtriangular incisions.

Aedeagus slender, parallel-sided, basally widened, apex rounded, in lateral view apex distinctly bent.

##### Etymology.

The name refers to the similarity with *Aplosonyxancora*.

##### Distribution.

China: Yunnan.

#### ﻿ Aplosonyx nigricornis sp. nov.

Taxon classificationAnimaliaColeopteraChrysomelidae

2BD9700B-679E-5391-8B3B-4E34438BAC71

https://zoobank.org/59E394F3-D8A9-47A7-83D2-EA75A9544D1C

Figs 29B , 30A–F

##### Type material.

***Holotype***: ♂, China, **Sichuan Province**, Qianjiang; 1750 m a. s. l.; 14 Jul. 1989; IZAS.

##### Diagnosis.

The new species closely resembles *A.nigriceps* but differs due to each elytron with five black spots in *A.nigriceps*; in this new species each elytron has six black spots, and the aedeagus apex is rounded.

##### Description.

**Male.** Length 5.0 mm, width 3.2 mm.

Head, antennae, pronotum, scutellum, ventral surface of thorax, abdomen, and legs black, elytra yellow, each elytron with six black spots, base, middle and apex with one pair of spots.

Vertex finely and sparsely covered with punctures. Interocular space 2.1 × as wide as transverse diameter of eye. Interantennal space 1.6 × as wide as transverse diameter of antennal socket. Frontal tubercles transverse, each separated by a deep furrow; antennae slender, 0.7 × as long as body; antennomeres 1–3 shiny; antennomeres 4–11 with short hairs, antennomere 2 shortest, antennomere 3 approximately 1.2 × as long as second; antennomere 4 longest, approximately 1.5 × as long as antennomeres 2 and 3 combined; antennomeres 5–10 gradually shortened, shorter than antennomere 4; antennomere 11 slightly longer than antennomere 10, pointed.

**Figure 30.**
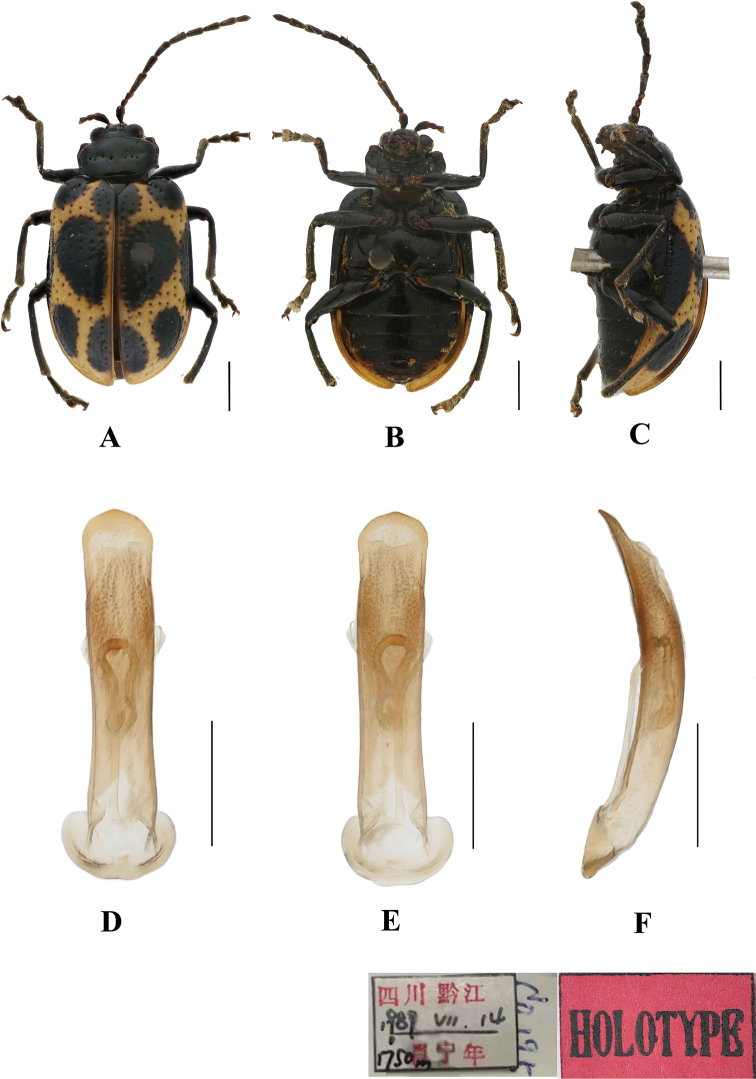
*Aplosonyxnigricornis* sp. nov. **A–C** habitus of holotype, IZAS**D–F** aedeagus **A, D** dorsal views **B, E** ventral views **C, F** lateral views. Scale bars 0.5 mm (**D–F**); 1 mm (**A–C**).

Pronotum 2 × as wide as long, lateral border margined, widest at anterior 1/3; disc with transverse furrow, less distinct in middle; covered with several large punctures in furrow and with sparsely small punctures in other parts of pronotum.

Scutellum triangular, only on base sparsely covered with small punctures.

Elytra wider than pronotum, 0.7 × as long as body, 1.65 × as long as wide, epipleura wide at anterior 1/3, posteriorly gradually narrowing towards apex, dorsal surface slightly convex, regularly covered with large punctures, partially arranged in ten rows in each elytron, the interstices of punctures in rows approximately 2.5 × as wide as the diameter of punctures and lightly covered with small punctures in interstices.

Metasternum 2 × as long as the mesosternum. Ventral surface of abdomen with five ventrites, ventrite 1 longest, ventrites 2–4 gradually shortened, apical ventrite slightly longer than ventrite 3, two subtriangular incisions.

Aedeagus slender, parallel-sided, basally widened, apically rounded, in lateral view distinctly bent.

##### Etymology.

The species name refers to the black antennal color.

##### Distribution.

China: Sichuan.

#### ﻿ Aplosonyx wudangensis sp. nov.

Taxon classificationAnimaliaColeopteraChrysomelidae

17EE4BA6-F7BB-5024-B6E9-CC5F0F50A5CC

https://zoobank.org/14B1F164-283A-4223-AAC4-BB505F9C5CD4

Figs 29C , 31A–F

##### Type material.

***Holotype***: ♂, China, **Hubei Province**, Wudang; 15 Aug. 1984; IZAS; IOZ(E)1566640.

##### Diagnosis.

The new species closely resembles *A.yunlongensis* in spots of elytra, but the pronotum of *A.yunlongensis* has three obvious black spots. The new species is different in that the black spots on both sides of pronotum are small and almost invisible. The new species also closely resembles *A.tianpingshanensis* in the aedeagus, where the apex is wide and flat in *A.tianpingshanensis*, while the new species is round and slightly pointed. The interstices of punctures on elytra of new species are narrower than that on the elytra of *A.yunlongensis* and *A.tianpingshanensis*.

##### Description.

**Male.** Length 5.0 mm, width 3.1 mm.

Head, antennae, pronotum, elytra and leg yellow, vertex, scutellum, and ventral surface of the body black, apical ventrite of abdomen yellow, pronotum with a black spot in middle, the black spots on both sides are small and almost invisible. each elytron with five black spots, middle and apex with one pair of spots and base with one spot.

Vertex sparsely covered with punctures. Interocular space 2.1 × as wide as transverse diameter of eye. Interantennal space 1.5 × as wide as transverse diameter of antennal socket. Frontal tubercles transverse, each separated by a deep furrow; antennae slender, 0.75 × as long as body; antennomeres 1–3 shiny; antennomeres 4–11 covered with pubescence, antennomere 2 shortest, antennomere 3 slightly longer than antennomere 2, approximately 1.4 × as long as second; antennomere 4 longest, approximately 1.2 × as long as antennomeres 2 and 3 combined; antennomeres 5–10 gradually shortened, shorter than antennomere 4; antennomere 11 slightly longer than antennomere 10, pointed.

**Figure 31.**
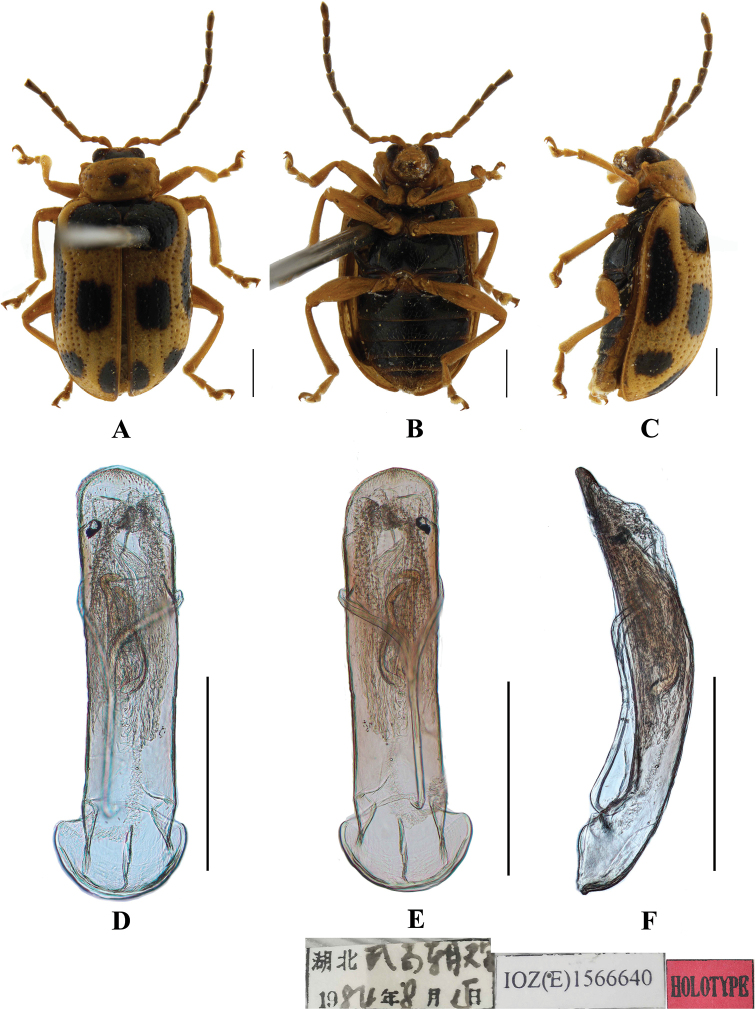
*Aplosonyxwudangensis* sp. nov. **A–C** habitus of holotype, IZAS IOZ(E)1566640 **D–F** aedeagus **A, D** dorsal views **B, E** ventral views **C, F** lateral views. Scale bars 0.5 mm (**D–F**); 1 mm (**A–C**).

Pronotum approximately 1.8 × as wide as long, lateral border margined, widest at anterior 1/3; disc with transverse furrow, the punctures are evenly distributed on the disc.

Scutellum triangular, smooth, impunctate.

Elytra: wider than pronotum, 0.8 × as long as body, 1.6 × as long as wide, epipleura wide at anterior 1/3, posteriorly gradually narrowing towards apex, dorsal surface slightly convex, regularly covered with large and deep punctures, partially arranged in ten rows in each elytron, the interstices of punctures lightly wider than diameter of individual punctures and covered with small punctures in the interstices.

Metasternum 2 × as long as the mesosternum. Ventral surface of abdomen with five ventrites, ventrite 1 longest, ventrites 2–4 gradually shortened, apical ventrite slightly longer than ventrite 3, two subtriangular incisions.

Aedeagus slender, parallel-sided, basally widened, apex rounded, in lateral view moderately bent.

##### Etymology.

This new species was named after the type locality.

##### Distribution.

China: Hubei.

## Supplementary Material

XML Treatment for Aplosonyx

XML Treatment for Aplosonyx ancora

XML Treatment for Aplosonyx chalybeus

XML Treatment for Aplosonyx cinctus

XML Treatment for Aplosonyx duvivieri

XML Treatment for Aplosonyx emeishanicus

XML Treatment for Aplosonyx flavipennis

XML Treatment for Aplosonyx fulvescens

XML Treatment for Aplosonyx gancuicus

XML Treatment for Aplosonyx nigriceps

XML Treatment for Aplosonyx omeiensis

XML Treatment for Aplosonyx orientalis

XML Treatment for Aplosonyx ornatus

XML Treatment for Aplosonyx pictus

XML Treatment for Aplosonyx robinsoni

XML Treatment for Aplosonyx rufipennis

XML Treatment for Aplosonyx sublaevicollis

XML Treatment for Aplosonyx tianpingshanensis

XML Treatment for Aplosonyx yunlongensis

XML Treatment for Aplosonyx ancorella

XML Treatment for Aplosonyx nigricornis

XML Treatment for Aplosonyx wudangensis
